# Multiplex Snapshot Minisequencing for the Detection of Common PAH Gene Mutations in Iranian Patients with Phenylketonuria

**DOI:** 10.52547/ibj.3856

**Published:** 2022-12-21

**Authors:** Pegah Namdar Aligoodarzi, Golale Rostami, Seyed Reza Kazemi Nezhad, Mohammad Hamid

**Affiliations:** 1 Department of Biology, Faculty of Science, Shahid Chamran University of Ahvaz, Ahwaz, Iran;; 2 Department of Molecular Medicine, Biotechnology Research Center, Pasteur Institute of Iran, Tehran, Iran

**Keywords:** Multiplex PCR, Mutation, Phenylalanine hydroxylase, Phenylketonurias

## Abstract

**Background:**

Phenylketonuria is a common inborn defect of amino acid metabolism in the world. This failure is caused by an autosomal recessive insufficiency of the hepatic enzyme PAH, which catalyzes the irreversible hydroxylation of phenylalanine to tyrosine. More than 1,040 different disease-causing mutations have already been identified in the *PAH* gene. The most prominent complication of PKU, if not diagnosed and treated, is severe mental retardation. Hence, early diagnosis and initiation of nutritional therapy are the most significant measures in preventing this mental disorder. Given these data, we developed a simple and rapid molecular test to detect the most frequent *PAH* mutations.

**Methods:**

Multiplex assay was developed based on the SNaPshot minisequencing approach to simultaneously perform genotyping of the 10 mutations at the *PAH* gene. We optimized detection of these mutations in one multiplex PCR, followed by 10 single-nucleotide extension reactions. DNA sequencing assay was also used to verify genotyping results obtained by SNaPshot minisequencing.

**Result:**

All 10 genotypes were determined based on the position and the fluorescent color of the peaks in a single electropherogram. Sequencing results of these frequent mutations showed that by using this method, a 100% detection rate could be achieved in the Iranian population.

**Conclusion:**

SNaPshot minisequencing can be useful as a secondary test in neonatal screening for HPA in neonates with a positive screening test, and it is also suitable for carrier screening. The assay can be easily applied for accurate and time- and cost-efficient genotyping of the selected SNPs in various population.

## INTRODUCTION

Phenylketonuria, the most common inborn defect of amino acid metabolism caused by a *PAH* deficiency, was first introduced by Asbjorn Folling in 1934^[^^1^^]^. The prevalence of this metabolic disease among whites has been reported as 1 in 10,000 and in the Iranian population as 1 in 3,627 live births^[^^2^^]^. PKU is the first known metabolic cause of mental retardation and the first genetic disorder of the central nervous system that can be fully treated by modification of external factors such as diet. PKU is also the first disorder successfully diagnosed by universal neonatal screening. Understanding the biochemical and molecular basis of PKU is of great importance in treatment strategies of this condition, resulting in a significant reduction in morbidity and an improvement in the quality of life^[^^3^^,^^4^^]^. Untreated PKU is linked to an atypical phenotype that includes intellectual disability, microcephaly, seizures, growth failure, and poor skin pigmentation. However, with early dietary intervention and the advent of newborn screening programs, PKU-positive infants can today be expected to have largely normal lives^[^^5^^,^^6^^]^. Mutations in the *PAH* gene on chromosome 12q23.2, are alterations that lead to the majority of PKU and HPA types^[^^7^^]^. Until now, over 1,040 disease-causing mutations have been reported^[^^8^^,^^9^^]^. The majority of these changes are corresponding to point mutations and cause missense mutations^[^^7^^,^^10^^]^.

Profound cognitive impairment caused by PKU is often managed by detecting the *PAH* deficiency in the newborn period (the first week of life) and prevented by initiating a specialized diet^[^^11^^]^. Genotyping, in most cases, is beneficial to the prediction of the phenotypic outcome as early as possible after birth. Besides, molecular confirmation is of significance in the diagnostic algorithm of HPA^[^^8^^,^^12^^]^. Sanger DNA sequencing, polymerase chain reaction-restriction fragment length polymorphism, and amplification-refractory mutation system are commonly used in research labs to identify SNPs^[^^13^^]^. Therefore, there is a need for a reliable, sensitive, and low-cost assay to detect numerous mutations in a single experiment as the assays are expensive and time-consuming in circumstances where several SNPs need to be examined^[^^14^^]^. The multiplex SNaPshot minisequencing assay uses a single-tube reaction to investigate SNPs at the specified locations. Its multiplex capabilities enable the analysis of more than 10 SNPs in a single reaction, regardless of their chromosomal locations or the distance between them and nearby SNP sites. Dideoxy single-base extension of an unmarked oligonucleotide primer(s) is/are the basis of its chemical reaction. Using DNA polymerase and a single suitable ddNTP that matches the nucleotide at the target site, a primer is hybridized to DNA close to a variant nucleotide site and extended using SNaPshot minisequencing. Capillary electrophoresis is used to separate and fluorescently detect the extended products^[^^15^^]^. The technique has demonstrated 100% sensitivity and 100% specificity, enabling researchers to use it as a quick confirmation test to achieve early genotyping, following a positive neonatal screening result. This genotyping test was successfully identified both alleles in PKU patients and it was quick and affordable. Considering these benefits, we developed a SNaPshot minisequencing method to detect ten common mutations, including, IVS2+5 G>C, IVS2-13 T>G, c.473G>A(p.R158Q), c.526C>T (p.R176*), c.691T >C (p.S231P), c.782G>A (p.R261Q), IVS 9+5 G>A, IVS 10-11 G>A, c.1068C>A( p.Y356*), and c.1208C>T (p.A403V). These point mutations are the most frequent reported mutant alleles in the studies carried out in different regions of Iran^[^^16^^,^^17^^,^^18^^-^^21^^]^. Except for IVS2+5, IVS2-13, IVS 9+5 and IVS 10-11, which are in intronic regions, the rest are in exonic regions. One of the selection criteria for the SNPs was their pathogenicity. All the selected SNPs are pathogenic variants that cause HPA in patients.

## MATERIALS AND METHODS

**Selection of SNPs**

There are no accurate statistics on the incidence of PKU in Iran. However, based on the reports, its prevalence in the country is about 0.027% (1 in 3,627 live births)^[^^22^^,^^23^^]^. Distribution of different types of *PAH* mutations varies in different regions of Iran. According to the studies carried out in various regions of Iran^[^^16^^,^^17^^,^^18^^-^^21^^]^, 10 common mutations with high frequencies were selected in our study (Table 1).

**Selection of samples**

Samples were selected from the patients referred to two PKU reference laboratories in Tehran and Ahwaz cities of Iran. The case files for 70 families of patients with suspected PKU phenotype were investigated to select 30 individuals with 10 common mutations. Three different individuals were chosen for each mutation as follows: one mutant homozygote, one normal homozygote, and one heterozygote. The salting out procedure was used to extract the genomic DNA from peripheral blood, which was then maintained at -20 °C for long-term storage^[^^24^^]^. A Nanodrop ND-1000 spectrophotometer was employed to evaluate the purity and concentration of DNA (NanoDrop Technologies, USA).

**Design of assay**

***Design of mPCR amplification primers***

Forward and reverse oligonucleotide primers were designed using Gene Runner (http://www.generunner. net/), primer 3 (https://primer3.ut.ee/), and NCBI primer blast (https://www.ncbi.nlm.nih.gov/tools/ primer-blast/) to amplify the DNA fragments encompassing the selected SNPs. Eight-pair primers were designed to sequence 10 SNPs (Table 2). In two cases, four SNPs (SNP4: rs199475575 and SNP5: rs5030845 as well as SNP8:rs5030855 and SNP9: rs62516095) were situated close to each other side by side in pairs (Table 1). Therefore, these two SNPs were included in one amplification target for each case. The lengths of the amplified genomic DNA segments ranged from 143 to 882 nucleotides. A total of 16 mPCR primers were designed based on the Ensemble reference sequence. All designed primers had a melting temperature between 58 and 63 ^o^C, (https://worldwide. promega.com/resources/tools/biomath/tm-calculator/), a purine:pyrimidine content of ~1:1, and lengths of 20-26 nucleotides. Using the BLAST program (http://blast.ncbi.nlm.nih.gov/Blast.cgi), the primer sequences were checked to avoid similarities with other loci or repetitive sequences in the genome, as well as confirm their specificity for the respective sequences. Secondary structures, hairpin structures, ΔΔG of self- and hetero-dimers, and potential primer-primer interactions were predicted using the OligoAnalyzer 3.1 tool (http://eu.idtdna.com/analyzer/ Applications/OligoAnalyzer/) and Gene Runner Version 6.3.03. Additionally, each designed primer was validated by SNP checker (https://genetools.org/ SNPCheck/snpcheck.htm) to investigate the possible presence of SNPs in their sequence. All mPCR primer sequences are listed in Table 2.

**Table 1:** *Selected *PAH* SNPs*

**Reference**	***PAH*** ** homos sequence**	**Nucleotide ** **position**	**Location ** **of SNP**	**NCBI reference SNP**	**SNP** **no.**
^[^^41^^,^^42^^]^	CGCTTATTTGAGGTCA[G>C]TGCTACAATCATGTTTGT	c.168+5G>C	IVS2+5 G>C	rs62507288	1
					
^[^^43^^]^	TGTCTCCTCACCCTCCCCA[T>G]TCTCTCTTCTAGGAGAATG	c.169-13T>G	IVS2-13 T>G	rs62507341	2
					
^[^^44^^]^	CCTGTGTACCGTGCAAGAC[G>A]GAAGCAGTTTGCTGACATT	c.473G>A	Exon 5 R158Q cGg>cAg	rs5030843	3
					
^[^^20^^,^^45^^]^	TGGGCAGCCCATCCCT[C>T]GAGTGGAATACATGGAGGAAG	c.526C>T	Exon 6 R176* C>T	rs199475575	4
					
^[^^21^^,^^45^^]^	CCCAGCTGGAAGACGTT[T>C]CTCAATTCCTGCAGAGT	c.691T>C	Exon 6 S231P Tct/Cct	rs5030845	5
					
^[^^41^^,^^42^^]^	TGGGTGGCCTGGCCTTCC[G>A]AGTCTTCCACTGCAC	c.782G>A	Exon 7 R261Q cGa>cAa	rs5030849	6
					
^[^^20^^]^	GAAAAGCTCGCCACAGTAA[G>A]TCCCTTCTCTCCCT	c.969 + 5G>A	IVS 9+5 G>A	rs62508637	7
					
^[^^41^^,^^42^^]^	GATAATAACTTTTCACTT[G>A]GGGCCTA	c.1066-11G>A	IVS 10-11 G>A	rs5030855	8
					
^[^^21^^]^	TTTCACTTGGGCCTACAGTA[C>A]TGCTTATCAGAGAAGCC	c.1068C>A	Exon 11 Y356* taC>taA	rs62516095	9
					
^[^^41^^,^^21^^]^	TTGGTCTTAGGAACTTTG[C>T]TGCCACAATACCTC	c.1208C>T	Exon 12, A403V gCt>gTt	rs5030857	10

***Minisequencing primer design***

Ten minisequencing primers were designed by Gene Runner software to identify each SNP from the eight amplified DNA fragments. Multiplex minisequencing primers were designed according to the Ensemble reference sequence. A primer melting temperature was calculated using the Biomaths Calculator program (https://worldwide.promega.com/resources/tools/biomath/tm-calculator/) by annealing one base before the SNP to be analyzed. Therefore, the polymorphisms would be identified by the base after the minisequencing primer for each SNP. The minisequencing products generated for the SNPs differ significantly in size and can easily be distinguished in a single capillary electrophoresis run^[^^25^^]^. Minisequencing primers were between 15 and 25 nucleotides in length with melting temperatures ranging from 49 to 53 ^o^C. The sequences of the primers were checked for possible hairpin structures or primer-dimer formation as described above and were further tested for self- extension in control PCRs without a template. In order to allow adequate separation of the primer-extension products in a single capillary electrophoresis, a non-homologous neutral sequence (a part or whole of a 40-nucleotide-long sequence [5’-aactgactaaactaggtgcc acgtcgtgaaagtctgacaa-3’]) was incorporated at the 5’-ends of the minisequencing primers to adjust their lengths and obtain a more balanced base composition^[^^26^^]^. The neutral sequence is a random sequence that does not match with any human sequence in the NCBI database^[^^27^^]^. The length of the primers was modified by adding a neutral sequence for all SNPs, except for Set-1. Their final lengths ranged from 17 to 80 nucleotides, and each product of the primer was spaced at least four nucleotides from the nearest primer product (Table 3). To improve electropherograms and inhibit the overlapping of nearby primers, which leads to nonspecific extensions, some primers were created on the reverse strand (marked with R in Table 3). 

**Table 2:** PAH* mPCR amplification primers*

**Product** **size (bp)**	**Concentration (µM)**	**Tm** ** (** ^o^ **C)**	**Primer** **length (nt)**	**mPCR primers (5′-3′)**	**Primer** **no.**
816	0.13	60	26	F: 5'-AGCTGATAACTGACCCAGTAGCATCA-3'	1
	0.13	60	25	R: 5'AGCTACTTGAAGAAAGGGACGGTGT-3'	
					
143	0.087	61	22	F: 5'-GACTGTCTCCTCACCCTCCCCA-3'	2
	0.087	62	22	R: 5'-TTGTCAGAGCAGGCAGGCTACG-3'	
					
178	0.13	59	22	F: 5'-ACTGTCATAGCTTGAGAGCCCC-3'	3
	0.13	58	20	R: 5'-AAGGCAGACTTACTGGCGGT-3'	
					
548	0.087	63	22	F: 5'-AGCTTGCACTGGTTTCCGCCTC-3'	4
	0.087	61	26	R: 5'-TGGCTGCAATTCTCTACCTCTACCCA-3'	
					
402	0.087	59	20	F: 5'-TGGCCACCCATCACCTTTTT-3'	5
	0.087	60	20	R: 5'-GCCTGTTGGTGGGTTCAAGA-3'	
					
286	0.13	60	20	F: 5'-TTCTTGGAGCCAGGGGACTA-3'	6
	0.13	62	20	R: 5'-ACTGAGAAGGGCCGAGGTAT-3'	
					
882	0.087	61	20	F: 5'-TGCATGCAGCCTTGTGAGTA-3'	7
	0.087	60	20	R: 5'-TGGTGGCAGGGATGAGTAGA-3'	
					
531	0.13	60	20	F: 5'-GATGGCAGCTCACAGGTTCT -3'	8
	0.13	61	20	R: 5'-CCACAGCCTCAGGTGTTTGA-3'	

F, forward primer; R, reverse primer; nt, nucleotide

**Multiplex PCR amplification**

Genotypic detection of *PAH* SNPs was performed using mPCR, followed by a multiplex SNaPshot minisequencing reaction. All amplicons were first tested in a singleplex PCR (not shown), and then eight pairs of multiplex primers were set up in one tube for convenience and low cost. Subsequently, a mix of eight primers was made, and then 3.5 µL of mix primer (final concentrations ranged 0.087-0.13 μM; Table 2), was added to 12.5 µL of PCR Master Mix. Next, 100 ng of genomic DNA was added to DNase/RNase-free distilled water and reached the final volume of 25 μL. PCRs were performed using TEMPase Hot Start Master Mix (Ampliqon, Denmark). Amplification was carried out in a thermocycler (Thermo Fisher Scientific, USA). After a pre-incubation step at 95 °C for 10 min, PCR was performed for a total of 30 cycles using the following conditions: denaturation at 95 °C for 30 s, annealing at 67 °C for 60 s, and extension at 72 °C for 60 s, followed by 10-min final extension at 72 °C.

**Multiplex SNaPshot reactions**

To set up minisequencing primers, exonuclease I and shrimp alkaline phosphatase enzymes were added to the mPCR products to clean the PCR product and remove single-stranded and non-specific sequences, excess primers, and unincorporated ddNTPs. PCR product (3 µL) with 1 µL of exonuclease I/shrimp alkaline phosphatase enzyme mixture (Affymetrix, Product no: 78200/01/02/05/50, USA) was combined and placed in a thermocycler. After 15 minutes at 37 °C, the reaction mixture was incubated at 80 °C for 15 min to inactivate the enzymes, after which they were kept at 4 °C until later use. 

**Table 3:** HPLC purified *PAH* minisequencing primers

**Primer name**	**SNP**	**Sequence (5'-3')**	**Primer** **length (nt)**	**Product** **size (bp)**	**Concentration** **(µM)**	**Tm** **(°C)**	**Gc (%)**
Set-1	rs62507288	F: GCGCTTATTTGAGGTCA	17	17	0.02	50	47
							
Set-2	rs62507341	F: acgtcgtgaaagtctgacaaTCCTCACC CTCCCCA	15	35	0.03	52	67
							
Set-3	rs5030843	F: gccacgtcgtgaaagtctgacaaTGTGTA CCGTGCAAGAC	17	40	0.06	52	53
							
Set-4	rs5030849	R: gacaaGTGTGCAGTGGAAGACT	17	22	0.04	51	53
							
Set-5	rs62508637	F: aggtgccacgtcgtgaaagtctgacaaGAA AAGCTCGCCACAGTAA	19	46	0.01	52	44
							
Set-6	rs5030857	F: aactgactaaactaggtgccacgtcgtgaaagt ctgacaaGGTTTTGGTCTTAGGAACTTTG	22	62	0.1	52	41
							
Set-7-1	rs5030855	F: actaggtgccacgtcgtgaaagtctgacaaT AACAGCGATAATAACTTTTCACTT	25	55	0.06	52	28
							
Set-7-2	rs62516095	R: aactgactaaactaggtgccttttttttttacgtcgtg aaagtctgtgaaagtctgacaaTTTGGCATCTCTGATAAGCA	20	80	0.4	52	40
							
Set-8-1	rs199475575	R: aactgactaaactaggtgccacgtcgtgaaagtct gtgaaagtctgacaaTTCCTCCATGTATTCCACTC	20	70	0.13	51	45
							
Set- 8-2	rs5030845	R: gtctgacaaACTTACTCTGCAGGAA TTGAG	21	30	0.06	52	43

F, forward primer; R, reverse primer

Through a fluorescent ddNTP, the reaction expands the minisequencing primers and generates unique products that are distinct to each SNP allele. On 10 independent control samples of DNA from whole blood used as controls, the minisequencing primers were first set up, individually. The reactions were optimized to define the best concentration and find the electropherograms without background noise, an ideal peak resolution and homogeneous peak height. Furthermore, the optimal amount of template genomic DNA was experimentally defined by testing different concentrations of DNA template for each Multiplex SNaPshot Mix. The test conditions were evaluated using DNAs for the known mutations. All 30 participants included in the test were analyzed for heterozygosity and homozygosity, and the electropherograms were re-evaluated for peak height, size, and resolution. Finally, the SNaPshot minisequencing reaction was performed in a 5-μL final volume using 1 μL of the treated PCR product, 2 μL of the minisequencing primer cocktail (final primer concentrations ranged 0.01-0.4 μM; Table 3), and 1 μL of SNaPshot® Multiplex Ready Reaction Mix (Applied Biosystems, Poland). The primer extension conditions consisted of 96 °C for 10 s, followed by 25 cycles for 10 s at 96 °C, 5 s at 50 °C, and 30 s at 60 °C and then kept at 4 °C until further use. Afterwards, the samples were treated with calf intestinal alkaline phosphatase (New England BioLabs, Whitby, Ontario, Canada) at 37 °C for 45 min, followed by 15 min at 75 °C for enzyme inactivation. The Multiplex SNaPshot reaction mix products (1 µL) were mixed with 8.8 µL of HiDi™ formamide (Thermo Fisher Scientific) and 0.2 µL of Gene Scan 120 LIZ as a size standard (Thermo Fisher Scientific). Capillary electrophoresis was undertaken on an ABI PRISM 3130XL Genetic Analyzer (Thermo Fisher Scientific) using POP-7 polymer. Multiplex extension products were visualized and analyzed automatically with GeneMapper™ Software (Thermo Fisher Scientific).

**Validation of the multiplex minisequencing assay**

Sanger sequencing was performed to confirm the multiplex minisequencing results (Supplementary Figs. 1-20).

## RESULTS

**Multiplex PCR product analysis**

**Fig. 1:**
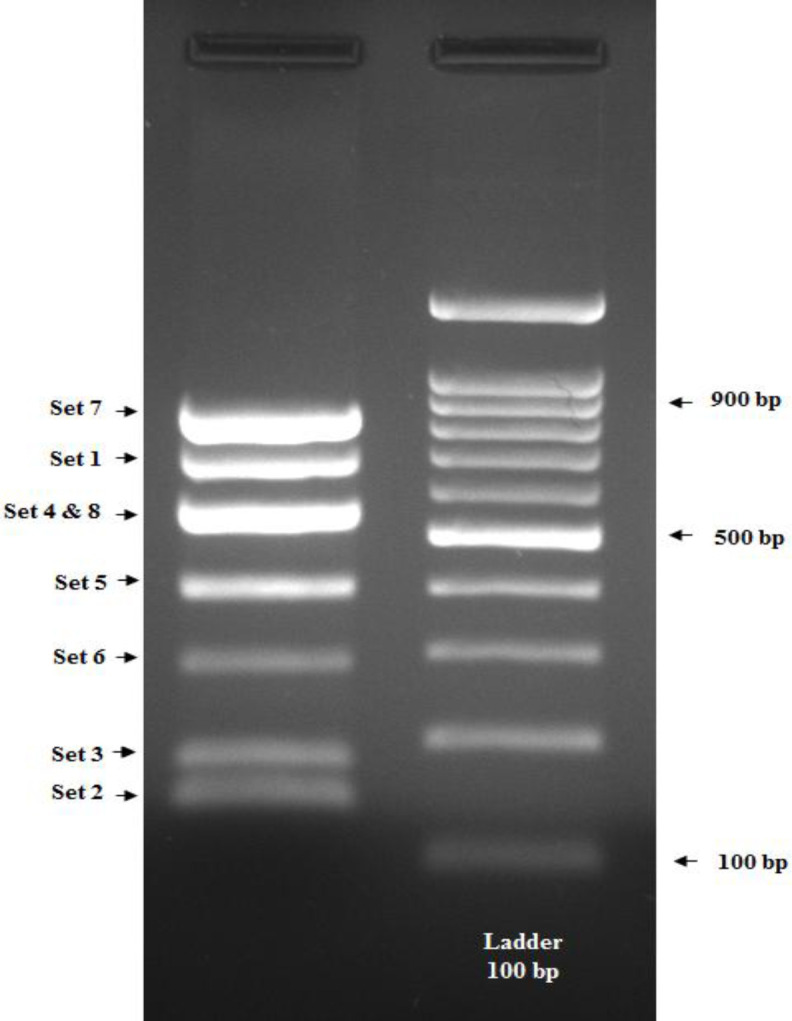
*Multiplex PCR amplified products. The agarose gel was stained with a green dye for visualizing the fragment migration.* *The left column is the mPCR product for eight primers, and the right column is the 100-bp DNA ladder*

The products of PCR reaction were analyzed by agarose gel electrophoresis using a 5-μL aliquot from the total reaction. The amplified products were run on a 1.5% agarose gel in a 0.5× Tris-Borate-EDTA solution at 90 v. The size of the products are listed in Table 2, and an example of *PAH *PCR amplified products is represented in Figure 1. Reaction products were stored at -20 °C until usage. 

**Minisequencing data analysis**

**Fig. 2:**
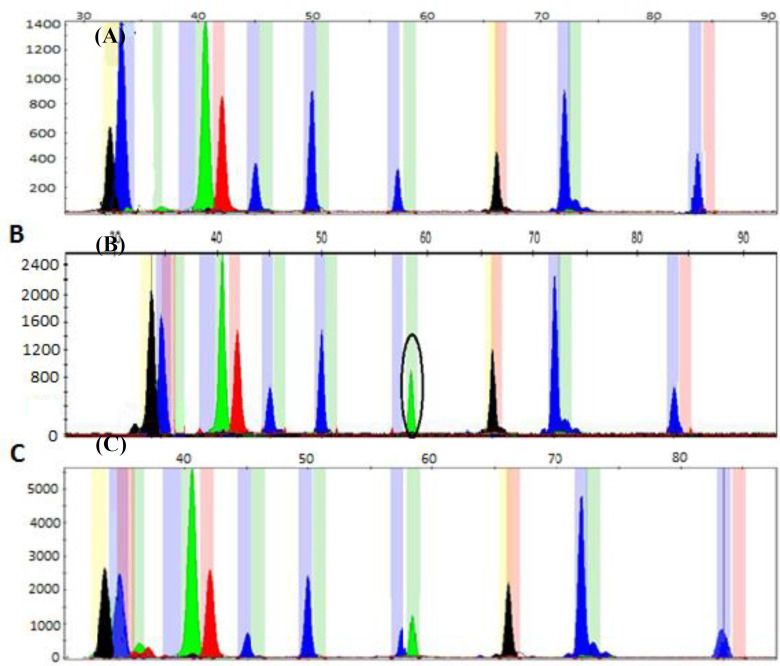
*Detection of *PAH* SNPs by multiplex minisequencing assays. The relative fluorescence units for the detected fragments as occurred over time are represented along the y-axis and size (nt) along the x-axis. (A) Multiplex minisequencing PCR results for a normal person for 10 primers. SNPs (detected genotype) from left to right, include: rs5030849, rs62507288, rs5030845, rs62507341, rs5030843, rs62508637, rs5030855, rs5030857,* *rs199475575, and rs62516095;* *(B) electropherogram indicates the multi-detection of *PAH* SNPs in a homozygote patient;* *As can be observed, even though the mutant homozygous and normal states of the minisequencing products* *have both the same size, the mutant peak is later than the normal peak due to the influence of dye on the mobility shift of DNA segments; (C) electropherogram indicating the multidetection of *PAH* SNPs in a heterozygote person*

**Fig. 3:**
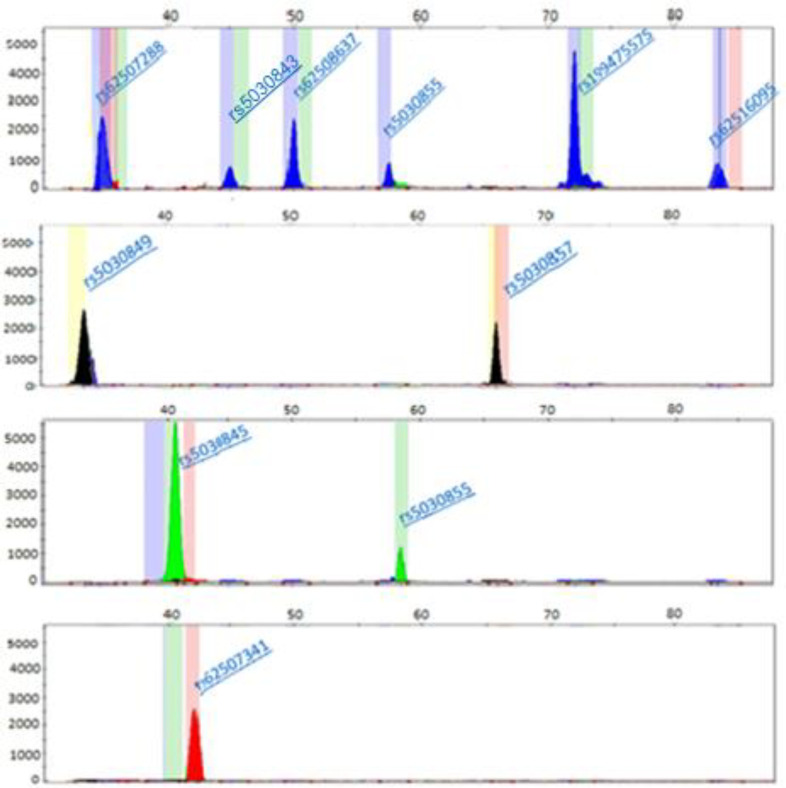
*Peaks on four separate electropherogram* *s* *based on their color, indicating the relative position of SNPs*. *The x-axis: the size (bp) of the prob pair with the incorporated nucleotides, the y-axis: the relative fluorescence unit of the peak*

We used multiplex minisequencing on the amplified PCR mixture of four terminator nucleotides (ddNTPs), each of which was labeled with a different fluorescent compound to find the point mutations in the *PAH *gene. One of the four dye terminators extended each primer molecule, and the fluorescent tag(s) was/were attached to the extended products. In minisequencing, a cycle sequencing reaction was carried out in the presence of Taq DNA polymerase, i.e. a mutation-detection primer was annealed such that its three nucleotide ends were before the mutation site, and a primer was served as a reporter of the wild-type and/or mutant genotype of the template DNA. Only a wild-type or mutant dye terminator was linked to the primer in wild-type or homozygous mutant samples. As a result, only one primer peak was shown on an electropherogram. However, in a heterozygous sample, both the wild-type and mutant dye terminators bind to the mutation detection primer, resulting in the detection of two distinct fluorescence signals or peaks. In addition, wild-type and mutant allele peak heights may significantly differ because of the differences in fluorescence emission of the fluorophores^[^^28^^]^ (Figs. 2 and 3). Data were analyzed by using the GeneMapper software. As each SNP minisequenced-treated fragment moved through the POP-7 polymer, its relative mobility to GeneScan 120LIZ size standards (15–120 nucleotide fragments) was used to determine its size^[^^25^^]^. The relative sizes and signal colors for each allele (major or minor) and SNP are shown in Table 4. The interval of 4, 8, and 9 nucleotide length differences between the neighboring minisequencing primers provided sufficient separation in the data after analysis.

**Validation of the multiplex minisequencing assay**

To estimate the precision of the assay, we screened the same cohort of patients sequenced for all 10 SNPs. The ratio of the correctly recognized SNPs (true positives), and the entity of any false-positive or false-negative peaks was tested. Our minisequencing results showed a 100% consistency with the genotypes defined by sequencing. No false-negative or false-positive results were detected. These data proved that the new developed multiplex minisequencing technique was highly accurate and appropriate for detecting 10 polymorphisms. 

## DISCUSSION

Identification of variants in the *PAH *gene is necessary to verify the diagnosis, select the treatment tactics, and detect the heterozygous carriers. In this regard, various studies have been conducted to identify the distribution of *PAH* gene mutations in different regions of Iran. In 2018, Esfahani et al.^[21]^ conducted a relatively complete study on the mutation spectrum of the *PAH* gene in the Iranian population. In that study, 34 different mutations were recognized with 100% mutation detection rate. IVS10-11G>A, p.P281L, R261Q, p.F39del, and IVS11+1G>C were the most prevalent mutations with frequencies of 26.07%, 19.3%, 12.86%, 6.07%, and 3.93%, respectively. All other mutations showed a relative frequency of less than 3.5%. That study was conducted on 140 Iranian patients with classic PKU. All important regions, including 13 exons as well as exon-intron boundaries of the *PAH* gene, were examined by direct DNA sequencing^[21]^. In a very recent study on the identification of the *PAH* gene mutation spectrum, a total of 129 different *PAH* gene mutations including, IVS10–11G>A (c.1066-11G>A; 19.23%), p.R261Q (c.782G>A; 7.63%), p.P281L (c.842C>T; 6.24%), IVS2 + 5G>C (c.168 + 5G>C; 5.75%), p.R243* (c.727C>T; 3.59%), IVS9+5G>A (c.969 + 5G>A; 2.84%), p.R176* (c.526C>T; 2.42%), p.Lys363Nfs*37 (c.1089delG; 2.13%), IVS11 + 1G>C (c.1199 + 1G>C; 2.07%), and p.L48S (c.143 T>C; 2.04%) were detected. That study was conducted on 1,547 patients with PKU^[^^20^^]^. In the two above-mentioned comprehensive studies, IVS10-11G>A, p.R261Q, and p.P281L mutations had a high rate, suggesting them as the dominant mutations in the Iranian population.

Detection of PKU by newborn screening and treatment at the beginning of the birth is a significant achievement in public health. The main method for detecting *PAH* gene mutations is DNA sequencing^[^^29^^,^^30^^]^ but Valian et al.^[^^31^^]^ used the PCR-RFLP method, and Bagheri et al.^[^^32^^]^ utilized *PAH* variable number of tandem repeat method. Also, a combination of single-strand conformation polymorphism and DNA sequencing methods was employed to identify *PAH* mutations^[^^2^^]^. These methods need to be confirmed with a precise technique such as DNA sequencing. Methods allowing simple, low-cost, fast, and high-throughput detection of mutations are becoming of particular interest in molecular diagnosis of genetic diseases. In one study, the minisequencing method was compared to sequencing and real-time PCR techniques^[^^33^^]^. 

Comparing the three techniques, including next-generation sequencing, SNaPshot, and real-time PCR showed the same results when the amount of DNA was sufficient. However, next-generation sequencing had a high cost^[^^33^^]^. SNaPshot technique has been also used to analyze human SNPs in criminological studies.

In this field, Snapshot technique has been mentioned as a cheap, versatile, and effective method^[^^34^^,^^35^^]^. The SNaPshot methodology was applied in 2014 to identify hepatitis B viral mutations, and the results revealed that this method is a reliable and affordable method for identifying the A-D genotype in this virus and is easily usable ^[^^36^^]^. Due to the high specificity of this technique to distinguish the sequence variants, it can be used in both research and routine laboratory operations for diagnosis, especially in criminology laboratories^[^^35^^,^^37^^,^^38^^]^. Our study confirmed the accuracy of the results and demonstrated the capability of this technique in detecting mutations, based on the sequencing technique. However, its advantages, in addition to lower cost, were great accuracy and rapid diagnosis. Thus, it can serve as a functional technique in clinical laboratories to identify either PKU mutations or mutations in the disorders such as thalassemia and mitochondrial diseases^[^^39^^]^. 

Based on the location and fluorescent color of the peaks on the electropherograms produced, we were able to detect the genotypes of the samples using the SNaPshot minisequencing approach. Based on the color and quantity of peaks at each mutation site in the electropherogram, minisequencing also successfully distinguished the heterozygosity from the homozygosity within the same reaction. The proper design of the multiplex minisequencing primers is essential for performance of the minisequencing assay. 

In the present study, we described a simple multiplex technique for simultaneous detection of 10 SNPs located in the *PAH* gene. The designed assays were based on the SNaPshot minisequencing method, which was found to be a precise assay for SNP genotyping in numerous biology fields, including forensic and population genetics^[^^14^^]^. The GeneMapper 1.6 software was employed to observe and analyze the peaks. Using GeneScan 120 LIZ size standards (15–120 nucleotide fragments) as a reference, each SNP minisequenced-treated fragment was given a size depending on how quickly it moved through the POP-7 polymer. A color was assigned to the individual dye-labeled ddNTP as follows: green/A, black/C, blue/G and red/T. The minisequencing reaction produced one (homozygote) or two (heterozygote) peaks depending on a SNP genotype. Homozygotes had only one peak either for the major allele or for the minor allele^[^^25^^]^. The relative sizes and signal colors for each allele (major or minor) of each SNP are shown in Table 4.

**Table 4:** *Relative size of the *PAH* SNaPshot products*

**Relative size (bp or nt)**	**Primers**	**SNP **	**Signal colors**	**SNP** **[major/minor]**	**Row**
32.5-33.5	Set4 mini-R	rs5030849	Black> Red	[C>T]	1
34-35.2	Set1 mini-F	rs62507288	Blue> Black	[G>C]	2
39.8-40.8	Set8-2 mini-R	rs5030845	Green> Blue	[A>G]	3
41.3-42.3	Set2 mini-F	rs62507341	Red> Blue	[T>G]	4
44.3-45.3	Set3 mini-F	rs5030843	Blue> Green	[G>A]	5
49.3-50.3	Set5 mini-F	rs62508637	Blue> Green	[G>A]	6
56.6-57.6	Set7-1 mini-F	rs5030855	Blue> Green	[G>A]	7
65.4-66.4	Set6 mini-F	rs5030857	Black> Red	[C>T]	8
71.5-72.5	Set8-1 mini-R	rs199475575	Blue> Green	[G>A]	9
83-84	Set7-2 mini-R	rs62516095	Blue> Red	[G>T]	10

* bP, base pair; nt, nucleotide; R, reverse primer; F, forward primer*

Although minisequencing reaction products for a specific SNP site had the same size, different electrophoretic mobilities of each incorporated dye-labelled ddNTP allowed visualization of two separate peaks and not two superimposed peaks of different colors^[^^25^^]^. The analyzed data showed a sufficient separation from the intervals of 4, 8, and 9 nucleotide length difference between the adjacent minisequencing primers. The stated sizes for each SNP will vary from the actual sizes (minisequencing primers sizes) by a few bases, since the dye affects the mobility shift of the DNA segments used in the POP-7. An example of a multiplex minisequencing electropherogram with 10 *PAH* detected SNPs for a normal homozygous, mutant homozygous and heterozygous individuals in rs5030855 is indicated in Figures 2 and 3. As represented in the two Figures, the height and color of the fluorescence peaks in 10 SNPs are different. In the normal person, according to the color of the displayed peaks, from left to right, the genotype of each SNP includes rs5030849: C/C, rs62507288: G/G, rs5030845: A/A, rs62507341: T/T, rs5030843: G/G, rs62508637: G/G, rs5030855: G/G, rs5030857: C/C, rs199475575: G/G, and rs62516095:G/G. In the mutant homozygous status for rs5030855, the genotype of the SNP includes A/A, and in the mutant heterozygote status for rs5030855, the genotype of the SNP entails G/A. Additionally, only one peak was observed in each site, and the genotype of all SNPs for a normal person was identified in a single reaction. However, two peaks were found in the SNP location for a person who was heterozygous in one SNP (Fig. 2C). Sometimes, depending on the type of mutation and the size of the primer, these two peaks overlapand the height of the peak is lower than that of the homozygous state.

As a method of polymorphism screening, the main advantage of the minisequencing assay is the simultaneous detection of many selected polymorphisms in a single reaction, with the results displayed in a single electropherogram. Each laboratory has a thermocycler and a DNA sequencer to perform the assay with ease. Noteworthy, this test would be useful in diagnostic labs having moderate to high patient sample volumes by using automated electrophoresis and subsequent data processing with the Genetic Analyzer device and Genotyper software^[^^14^^]^.

Using this technique for simultaneous detection of several polymorphisms at distant genetic locations in a single reaction, makes it cost- and time-efficient compared to other commonly used genotyping assays such as PCR-RFLP and Sanger DNA sequencing. Although PCR-RFLP is a common approach, genotyping the high number of SNPs selected for the two loci requires many PCR reactions, which is time-consuming and impractical. More crucially, because some SNPs under research have no useful restriction enzyme recognition sites or have additional SNPs inside the recognition site, PCR-RFLP is not applicable for all SNPs under investigation^[^^40^^]^. While it is possible to screen large-scale sequences or SNPs using next-generation sequencing and arrays, these high-throughput techniques are expensive and require the specialized equipment, that are not commonly available in most molecular biology laboratories. In addition, the minisequencing assays can be combined with multiplex ligation-dependent probe amplification technique or Gap-PCR for rapid detection of the deletions or duplications^[^^14^^]^. In conclusion, our method can be useful as a secondary test in neonatal screening for HPA to identify all common mutations in Iranian patients with a positive screening test. It is also a suitable method for carrier screening.

## DECLARATIONS

### Acknowledgments

We acknowledge the help and collaboration of the patients for their participation in the study. 

### Ethical statement

The study protocol was approved by the Ethical Committee of the Pasteur Institute of Iran, Tehran (ethical code: IR.PII.REC.1400.033). Informed consent was obtained from adult subjects and the parents of minors for being included in the study.

### Data availability

The raw data supporting the conclusions of this article are available from the authors upon reasonable request. 

### Author contributions

PNA: performed experiments and wrote manuscript; GR: conducted experiments and assisted in the final revision of manuscript; SRK: assisted in the final revision of manuscript; MH: designed the study and analyzed the data.

### Conflict of interest

None declared.

### Funding/support

This study was supported by the Pasteur Institute of Iran (grant number 815).

## Supplementary Materials

Supplementary Figs. 1-20

## References

[1] Christ SE (2003). Asbjorn folling and the discovery of phenylketonuria. Journal of the history of the neurosciences.

[2] Zare Karizi S, Hosseini Mazinani S, Khazaei Koohpar Z, Seifati S, Shahsavan Behboodi B, Akbari M, Koochmeshgi J (2011). Mutation spectrum of phenylketonuria in Iranian population. Molecular genetics and metabolism.

[3] Elhawary NA, Aljahdali IA, Abumansour IS, Elhawary EN, Gaboon N, Dandini M, Madkhali A, Alosaimi W, Alzahrani A, Aljohani F, Melibary EM, Kensara OA (2022). Genetic etiology and clinical challenges of phenylketonuria. Human genomics.

[4] Muntau AC, Gersting SW (2010). Phenylketonuria as a model for protein misfolding diseases and for the development of next generation orphan drugs for patients with inborn errors of metabolism. Journal of inherited metabolic disease.

[5] Blau N, Harding C, Burlina A, Longo N, Bosch AM (2021). Phenylketonuria. Nature reviews.Disease primers.

[6] Williams RA, Mamotte CD, Burnett JR (2008). Phenylketonuria: an inborn error of phenylalanine metabolism. The Clinical biochemist. Reviews.

[7] Pronina N, Giannattasio S, Lattanzio P, Lugovska R, Vevere P, Kornejeva A (2003). The molecular basis of phenylketonuria in Latvia. Human mutation.

[8] Tolve M, Artiola C, Pasquali A, Giovanniello T, D’Amici S, Angeloni A, Pizzuti A, Carducci C, Leuzzi D, Carducci C (2018). Molecular analysis of PKU-associated PAH mutations: a fast and simple genotyping test. Methods and protocols.

[9] Blau N, Yue W, Perez B PAHvdb. 2006–2017.

[10] Li N, He C, Li J, Tao J, Liu Z, Zhang C, Yuan Y, Jiang H, Zhu J, Deng Y, Guo Y, Li Q, Yu P, Wang Y (2018). Analysis of the genotype-phenotype correlation in patients with phenylketonuria in mainland China. Scientific reports.

[11] Brosco JP, Paul DB (2013). The political history of PKU: reflections on 50 years of newborn screening. Pediatrics.

[12] Zschocke J, Haverkamp T, Møller LB (2012). Clinical utility gene card for: Phenylketonuria. European journal of human genetics.

[13] Fateh A, Aghasadeghi M, Siadat SD, Vaziri F, Sadeghi F, Fateh R, Keyvani H, Tasbiti AH, Yari S, Ataei Pirkooh A, Monavari SA (2016). Comparison of three different methods for detection of IL28 rs12979860 polymorphisms as a predictor of treatment outcome in patients with hepatitis C virus. Osong public health and research perspectives.

[14] Fanis P, Kousiappa I, Phylactides M, Kleanthous M (2014). Genotyping of BCL11A and HBS1L-MYB SNPs associated with fetal haemoglobin levels: a SNaPshot minisequencing approach. Osong public health and research perspectives.

[15] Jian Y, Li M (2021). A narrative review of single-nucleotide polymorphism detection methods and their application in studies of Staphylococcus aureus. Journal of Bio-X research..

[16] Ganji F, Naseri H, Rostampour N, Sedighi M, Lotfizadeh M (2018). Assessing the phenylketonuria screening program in newborns, Iran 2015-2016. Acta medica Iranica.

[17] Biglari A, Saffari F, Rashvand Z, Alizadeh S, Najafipour R, Sahmani M (2015). Mutations of the phenylalanine hydroxylase gene in Iranian patients with phenylketonuria. Springerplus.

[18] Moghadam MR, Shojaei A, Babaei V, Rohani F, Ghazi F (2018). Mutation analysis of phenylalanine hydroxylase gene in Iranian patients with phenylketonuria. Medical journal of the Islamic Republic of Iran.

[19] Hamzehloei T, Hosseini S, Vakili R, Mojarad M (2012). Mutation spectrum of the PAH gene in the PKU patients from Khorasan Razavi province of Iran. Gene.

[20] Alibakhshi R, Mohammadi A, Salari N, Khamooshian S, Kazeminia M, Moradi K (2021). Spectrum of PAH gene mutations in 1547 phenylketonuria patients from Iran: a comprehensive systematic review. Metabolic brain disease.

[21] Esfahani MS, Vallian S (2019). A comprehensive study of phenylalanine hydroxylase gene mutations in the Iranian phenylketonuria patients. European journal of medical genetics.

[22] Mojibi N, Ghazanfari Sarabi S, Hashemi Soteh SMBH (2021). The Prevalence and incidence of congenital phenylketonuria in 59 countries: A systematic review. Journal of pediatrics review.

[23] Bagheri M, Rad IA, Jazani NH, Zarrin R, Ghazavi A (2015). Mutation analysis of the phenylalanine hydroxylase gene in Azerbaijani population, a report from West Azerbaijan province of Iran. Iranian journal of basic medical sciences.

[24] Miller SA, Dykes DD, Polesky HF (1988). A simple salting out procedure for extracting DNA from human nucleated cells. Nucleic acids research.

[25] Fiorentino F, Magli M, Podini D, Ferraretti A, Nuccitelli A, Vitale N, Baldi M, Gianaroli L (2003). The minisequencing method: an alternative strategy for preimplantation genetic diagnosis of single gene disorders. Molecular human reproduction.

[26] Sanchez JJ, Borsting C, Hallenberg C, Buchard A, Hernandez A, Morling N (2003). Multiplex PCR and minisequencing of SNPs—a model with 35 Y chromosome SNPs. Forensic science international.

[27] Lindblad Toh K, Winchester E, Daly MJ, Wang DG, Hirschhorn JN, Laviolette JP, Reich D E, Robinson E, Sklar P, Shah N, Thomas D, Fan J B, Gingeras T, Warrington J, Patil N, Hudson T J, Lander ES (2000). Large scale discovery and genotyping of single-nucleotide polymorphisms in the mouse. Nature genetics.

[28] Wang W, Kham SKY, Yeo G, Quah T, Chong SS (2003). Multiplex Minisequencing Screen for Common Southeast Asian and Indian Thalassemia Mutations. Clinical chemistry.

[29] Moradi K, Alibakhshi R, Ghadiri K, Khatami SR, Galehdari H (2012). Molecular analysis of exons 6 and 7 of phenylalanine hydroxylase gene mutations in Phenylketonuria patients in Western Iran. Indian journal of human genetics.

[30] Alibakhshi R, Moradi K, Mohebbi Z, Ghadiri K (2014). Mutation analysis of PAH gene in patients with PKU in western Iran and its association with polymorphisms: identification of four novel mutations. Metabolic brain disease.

[31] Vallian S, Barahimi E, Moeini H (2003). Phenylketonuria in Iranian population: a study in institutions for mentally retarded in Isfahan. Mutation research.

[32] Bagheri M, Rad IA, Jazani NH, Zarrin R, Ghazavi A (2014). Association between PAH mutations and VNTR alleles in the West Azerbaijani PKU patients. Maedica.

[33] Cernomaz A T, Macovei II, Pavel I, Grigoriu C, Marinca M, Baty F, Peter S, Zonda R, Brutsche M, Grigoriu BD (2016). Comparison of next generation sequencing, SNaPshot assay and real-time polymerase chain reaction for lung adenocarcinoma EGFR mutation assessment. BMC pulmonary medicine.

[34] Ralf A, Oven M Van, Montiel D, Knij P De, Beek K Van Der, Wootton S, Lagacé R, Kayser M (2019). Forensic Science International : Genetics Forensic Y-SNP analysis beyond SNaPshot : High-resolution Y-chromosomal haplogrouping from low quality and quantity DNA using Ion AmpliSeq and targeted massively parallel sequencing. Forensic science international. genetics.

[35] Mehta B, Daniel R, Phillips C, McNevin D (2017). Forensically relevant SNaPshot assays for human DNA SNP analysis: a review. International journal of legal medicine.

[36] Lai G, Zhang W, Tang H, Zhao T, Wei L, Tao Y, Wang Z, Huang A (2014). A SNaPshot assay for the rapid and simple detection of hepatitis B virus genotypes. Molecular medicine reports.

[37] Arpan A, Salil V, Harsh P, Nm P (2016). Single base primer extension assay (SNaPshot) for rapid detection of human immunodeficiency virus 1 drug resistance mutations. journal of molecular biomarkers and diagnosis.

[38] Samples R (2015). Genetics and genome research A comparison of SNaPshot minisequencing and HRM analysis in mtSNP clinMed. Journal of genetics and genome research.

[39] Fanis P, Kousiappa I, Phylactides M, Kleanthous M (2014). Minisequencing assay for the detection of 12 BCL11A single nucleotide polymorphisms. BMC genomics.

[40] Hashim HO, Al Shuhaib MBS (2019). Exploring the potential and limitations of PCR-RFLP and PCR-SSCP for SNP detection: A review. Journal of applied biotechnology reports.

[41] Gundorova P, Stepanova AA, Kuznetsova IA, Kutsev SI, Polyakov A V (2019). Genotypes of 2579 patients with phenylketonuria reveal a high rate of BH4 non-responders in Russia. PLoS one.

[42] Shaykholeslam Esfahani M, Shaykholeslam Esfahani E, Vallian S (2018). A novel compound-primed multiplex ARMS-PCR (CPMAP) for simultaneous detection of common PAH gene mutations. Metabolic brain disease.

[43] Tresbach RH, Sperb-Ludwig F, Ligabue-Braun R, Tonon T, de Oliveira Cardoso MT, Heredia RS, Teresa Alves da Silva Rosa M, Cátia Martins B, Oliveira Poubel M, Carlos Santana da Silva L, Maillot F, Doederlein Schwartz V (2021). Phenylketonuria diagnosis by massive parallel sequencing and genotype-phenotype association in brazilian patients. Genes (Basel).

[44] Zarinkoob M, Khazaei Koohpar Z (2022). Mutation analysis of exon 5 of PAH gene in phenylketonuria patients from Golestan Province, Iran. Journal of Shahrekord university of medical sciences..

[45] Jafarzadeh Esfehani R, Vojdani S, Hashemian S, Mirinezhad M, Pourafshar M, Forouzanfar N, Zargari S, Ehsan Jaripour M, Sadr Nabavi A (2020). Genetic variants of the phenylalanine hydroxylase gene in patients with phenylketonuria in the northeast of Iran. Journal of pediatric endocrinology and metabolism.

