# Supplementary material for: Multiplex Snapshot Minisequencing for the Detection of Common PAH Gene Mutations in Iranian Patients with Phenylketonuria
Source: Iran Biomed J. 2022 Dec 21;27(1):46–57. doi: 10.52547/ibj.3856 (PMC9971712; doi:10.52547/ibj.3856)
Supplement: Supplementary file 1 — Supplementary Figs. 1-20 [file ibj-27-46-s1.pdf]

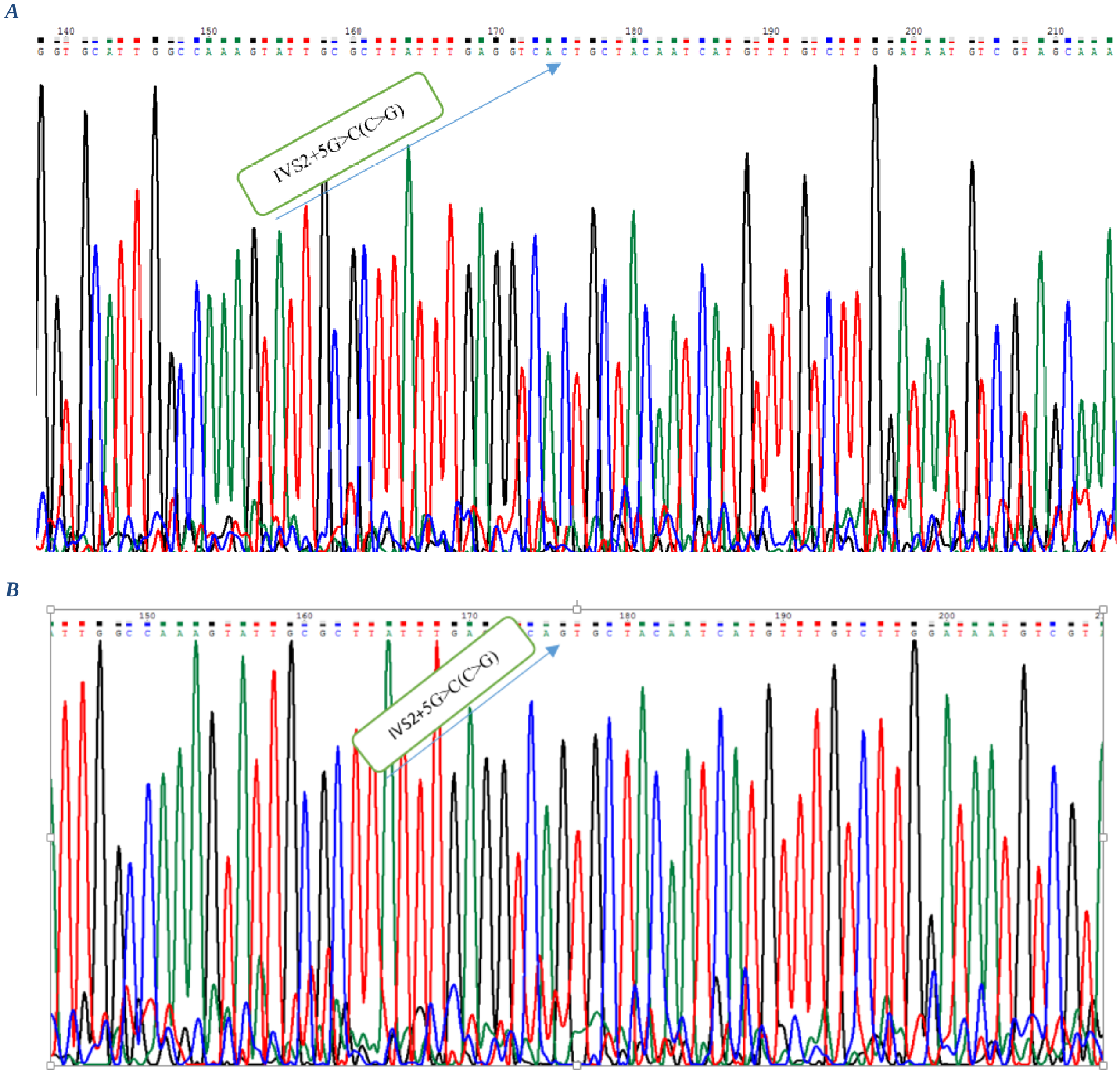

Supplementary Fig. 1. Homozygous (A) mutant and (B) normal for IVS2+5G>C (C>G) (forward strand).

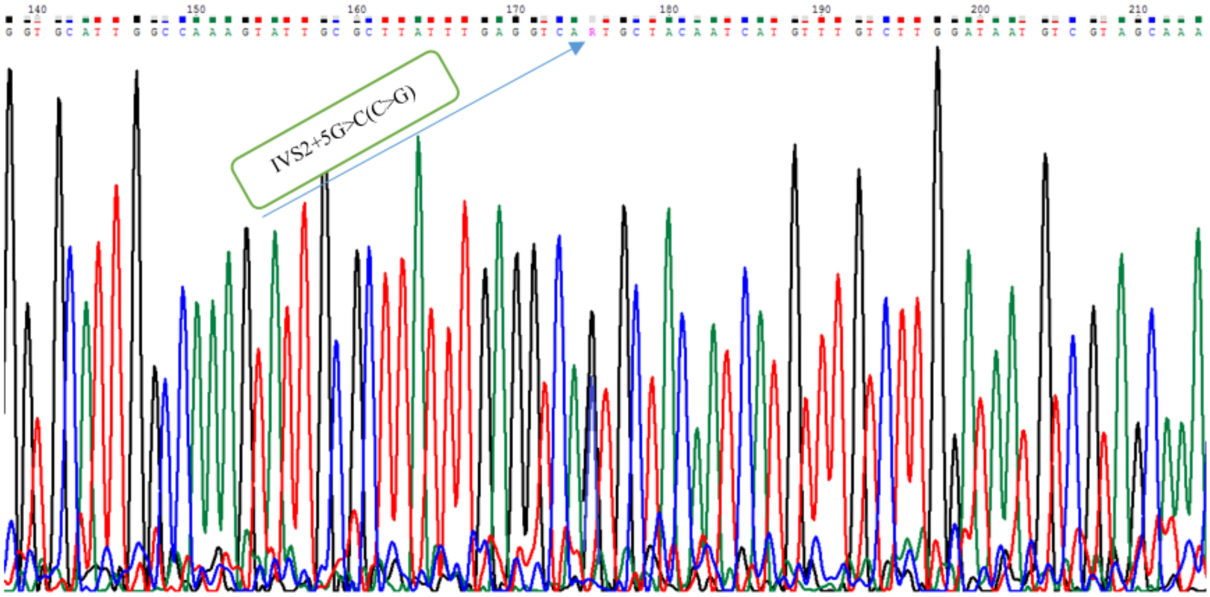

Supplementary Fig. 2. Heterozygous for IVS2+5G>C(C>G) (forward strand).

A

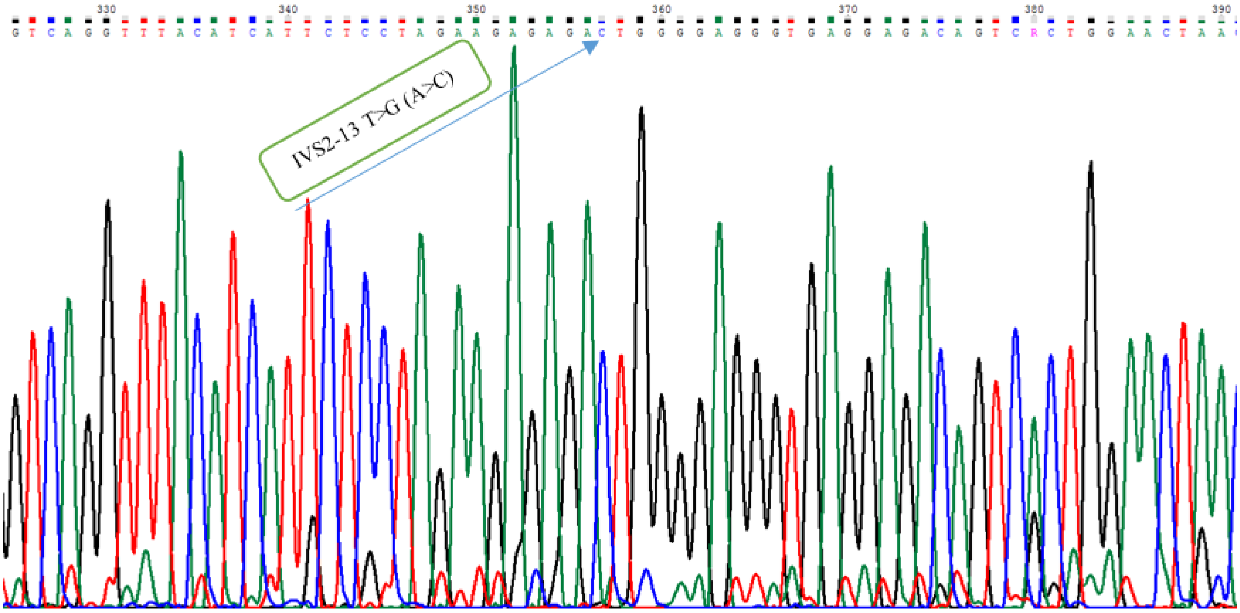

B

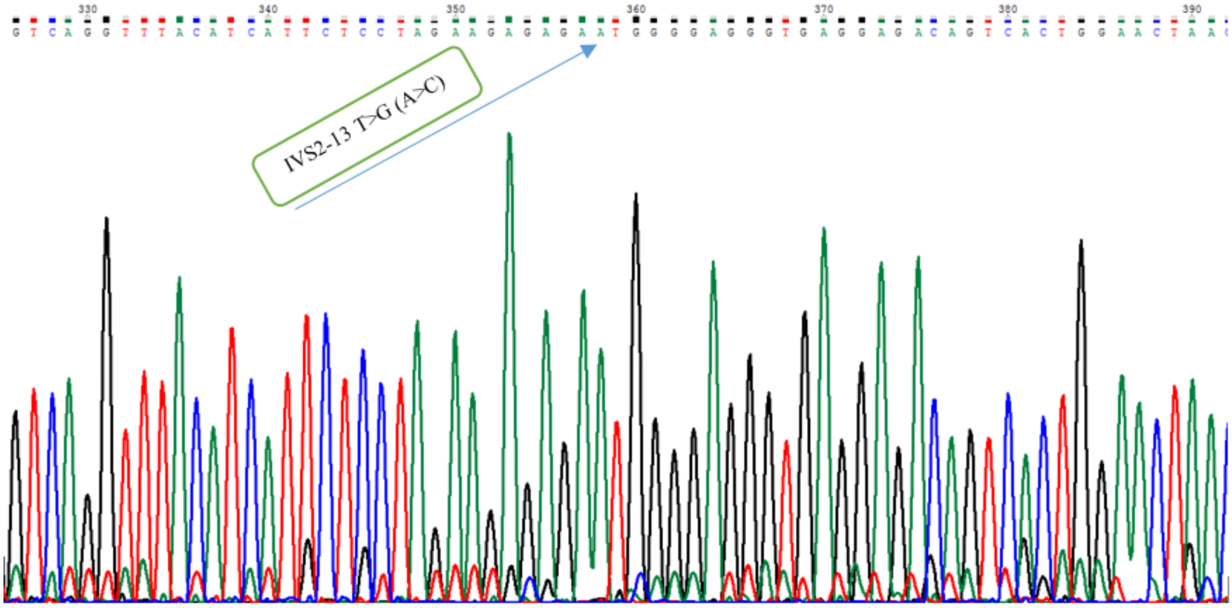

Supplementary Fig. 3. Homozygous (A) mutant and (B) normal for IVS2-13 T>G (A>C) (reverse strand).

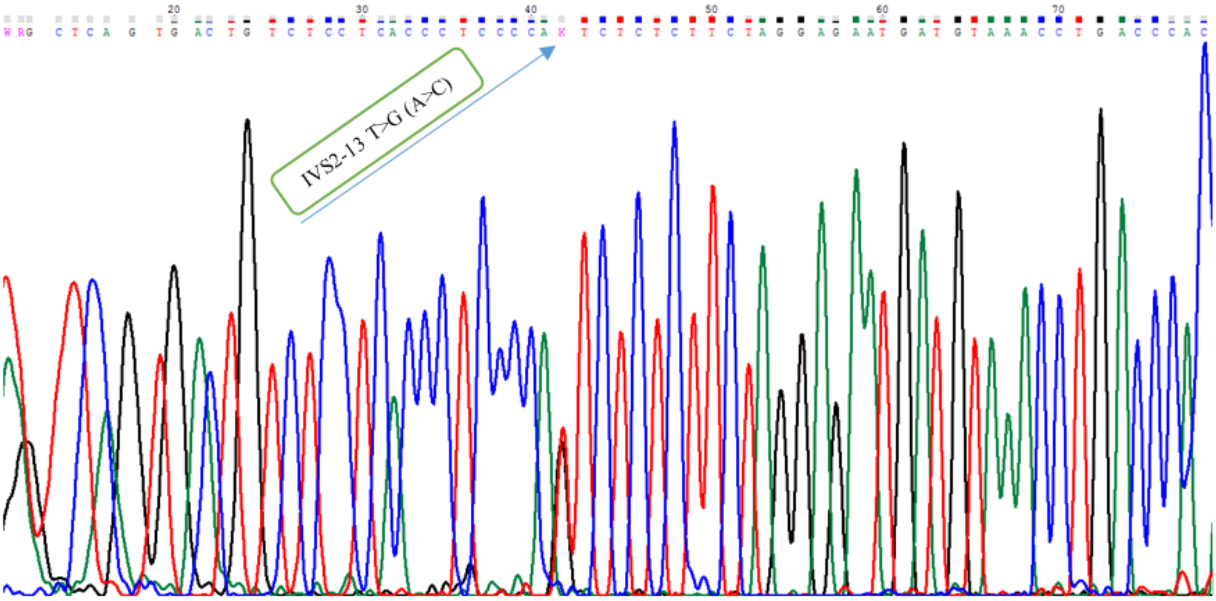

Supplementary Fig.4. Heterozygous for IVS2-13 T>G (A>C) (forward strand).

A

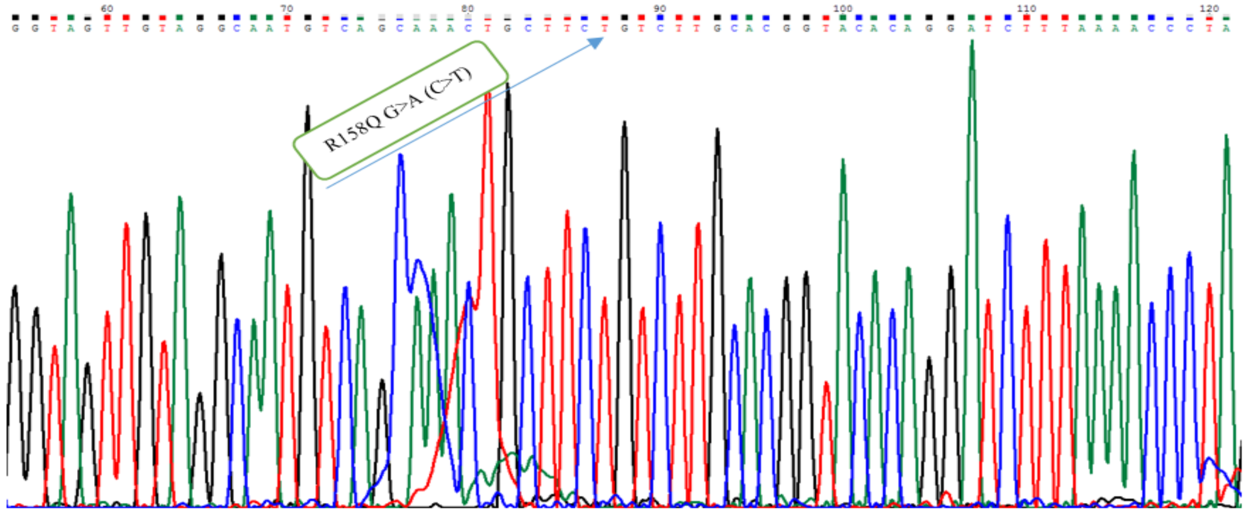

B

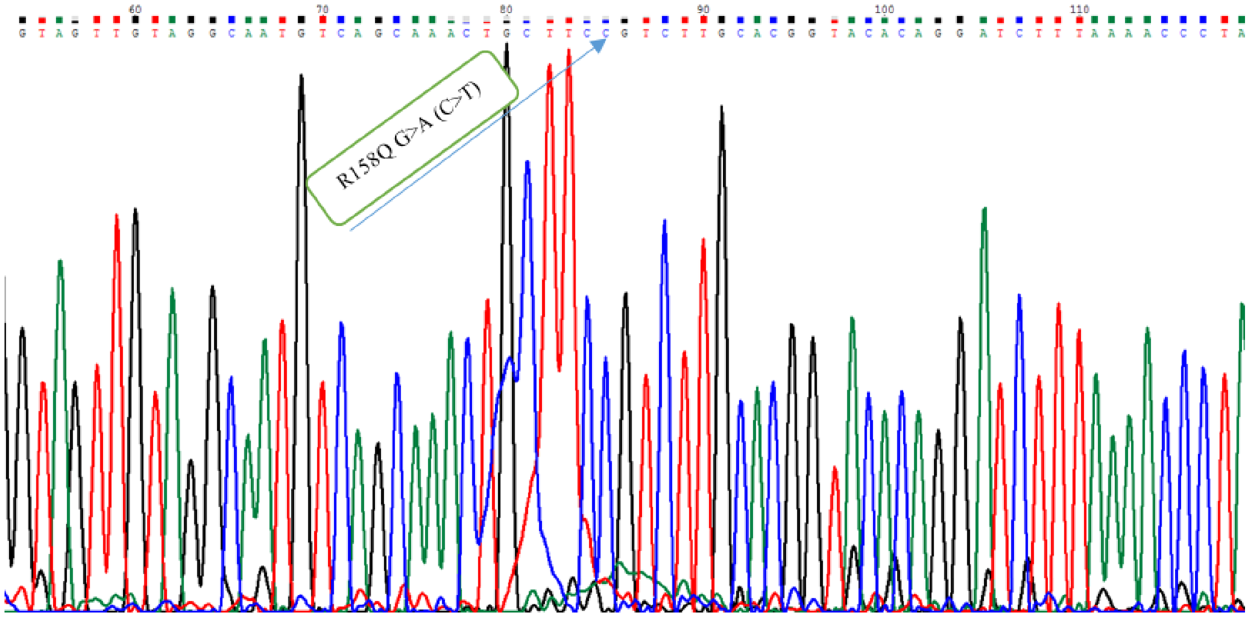

Supplementary Fig. 5. Homozygous (A) mutant and (B) normal for R158Q G>A(C>T) (reverse strand).

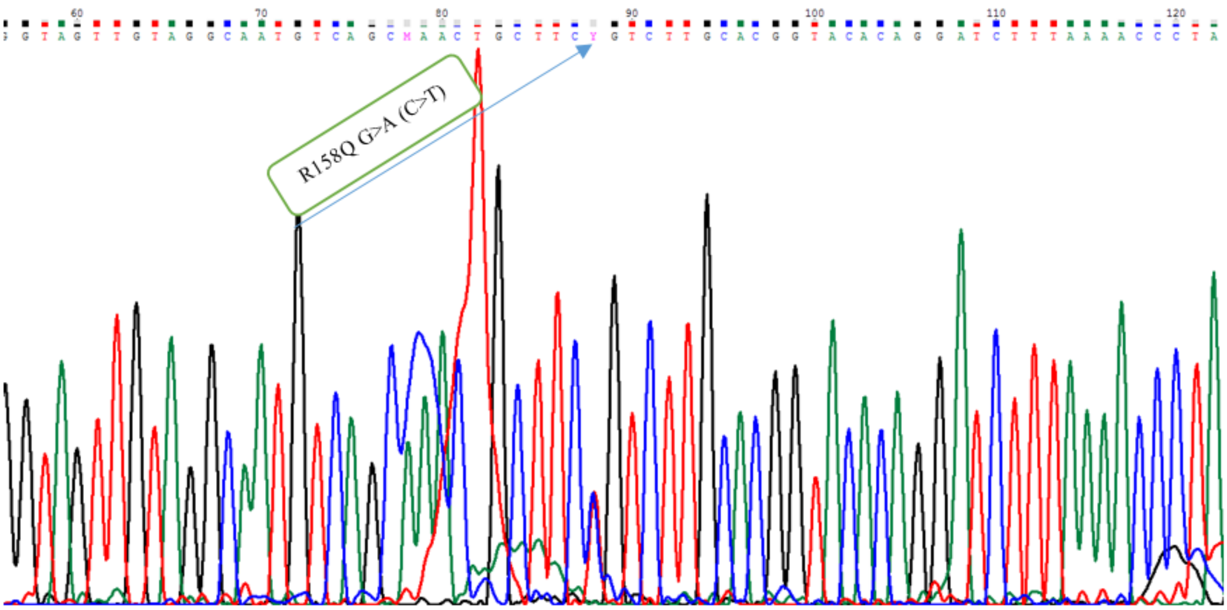

Supplementary Fig. 6. Heterozygous for R158Q G>A(C>T) (reverse strand).

A

B

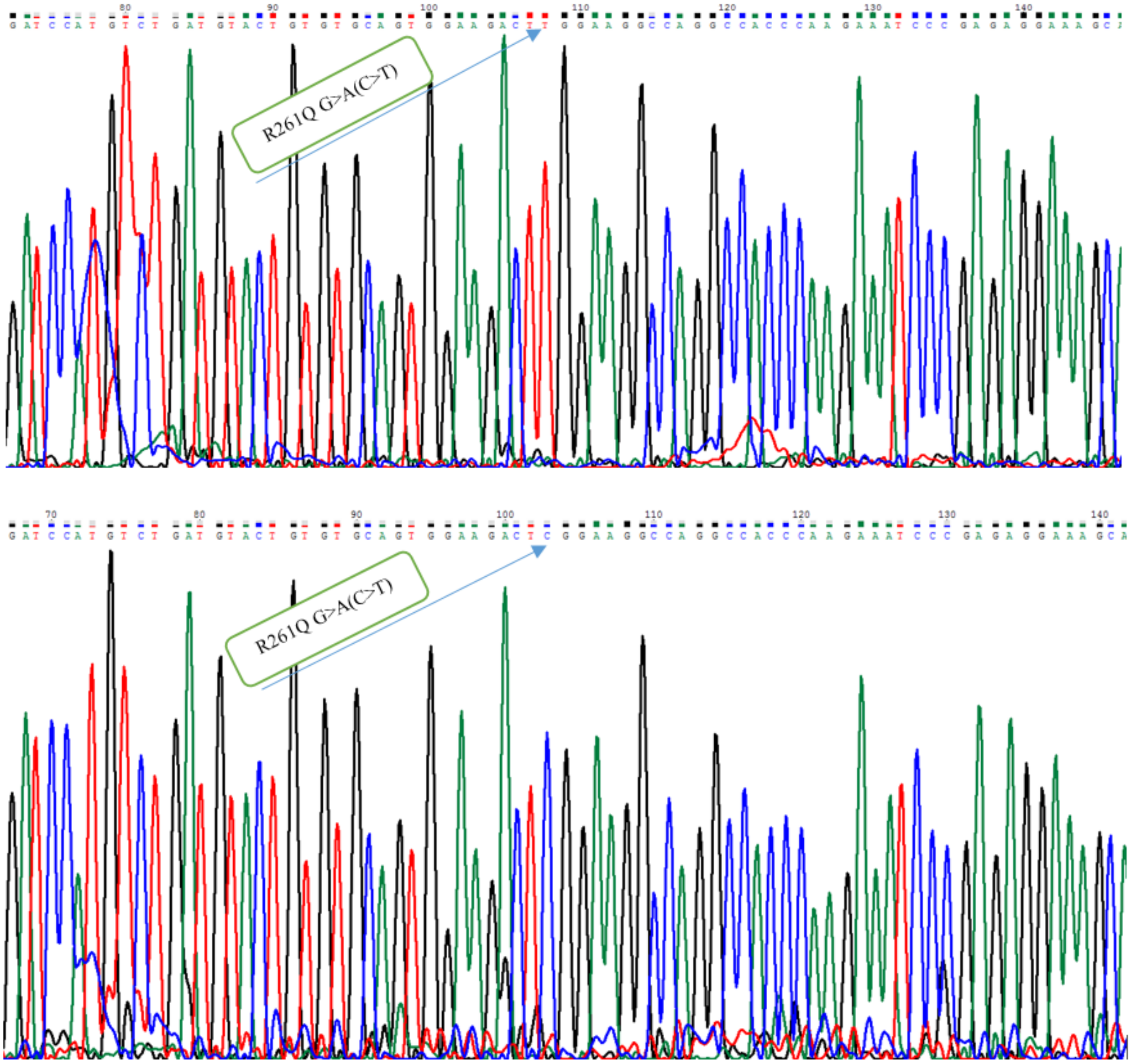

Supplementary Fig. 7. Homozygous (A) mutant and (B) normal for R261Q G>A(C>T) (reverse strand).

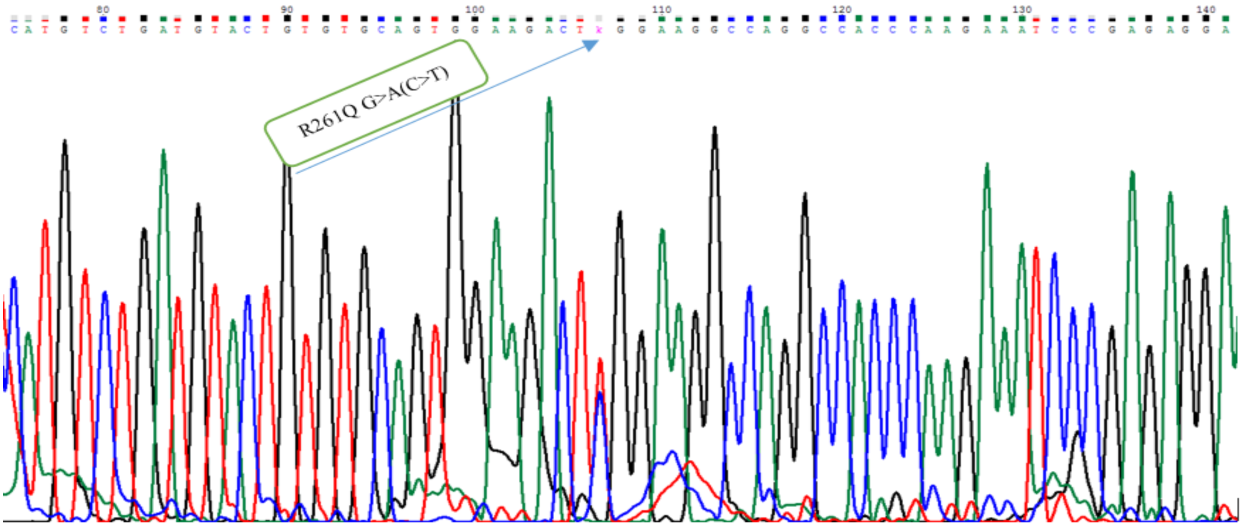

Supplementary Fig. 8. Heterozygous for R261Q G>A(C>T) (reverse strand).

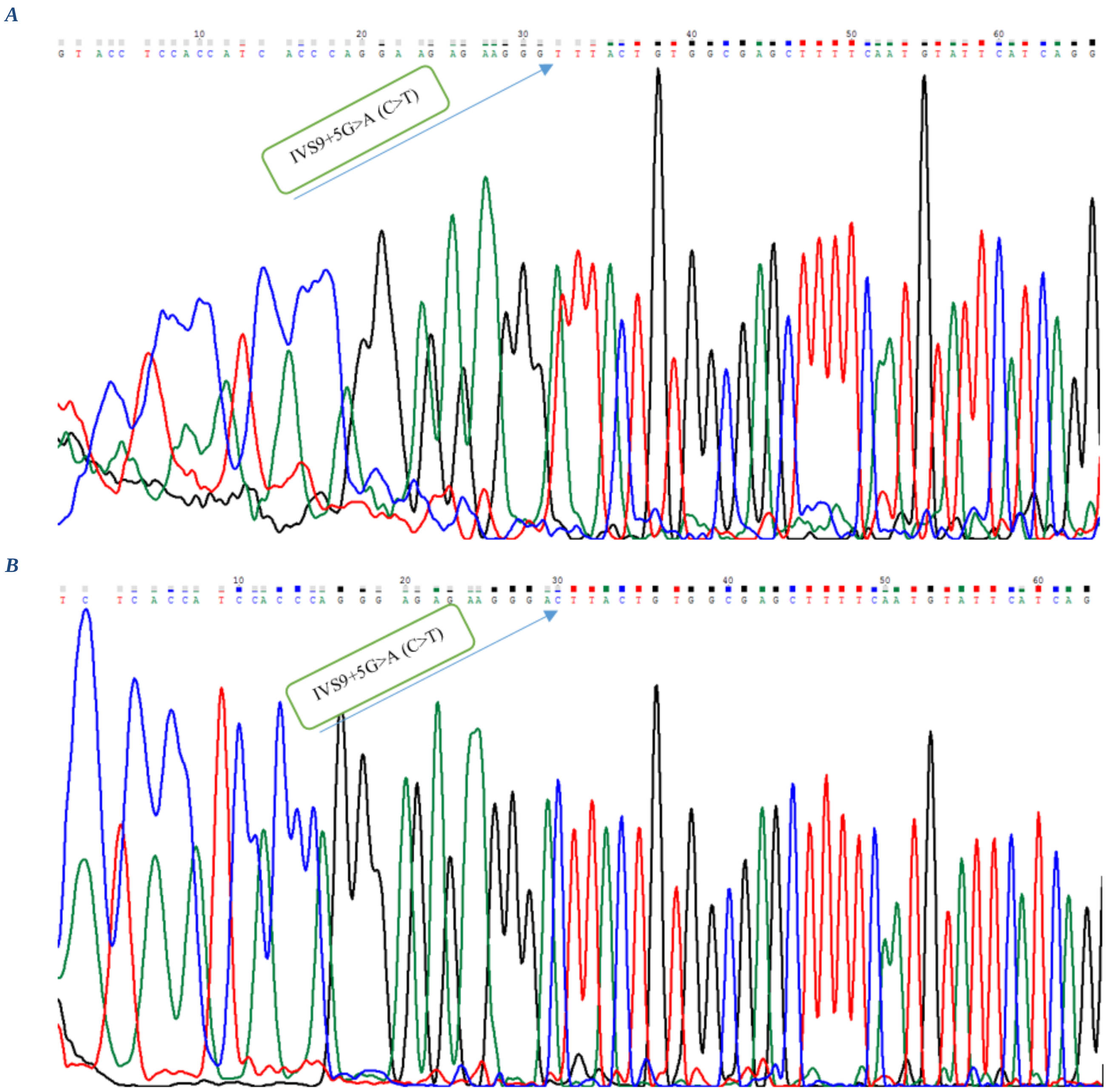

Supplementary Fig. 9. Homozygous (A) mutant and (B) normal for IVS9+5G>A (C>T) (reverse strand).

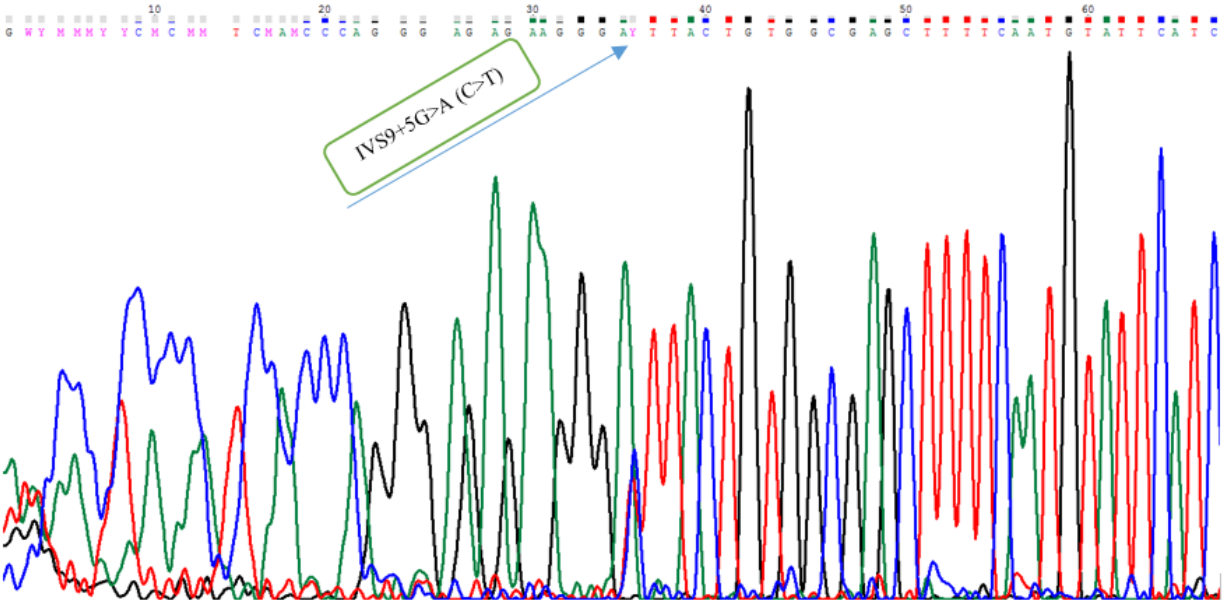

Supplementary Fig. 10. Heterozygous for IVS9+5G>A(C>T) (reverse strand).

A

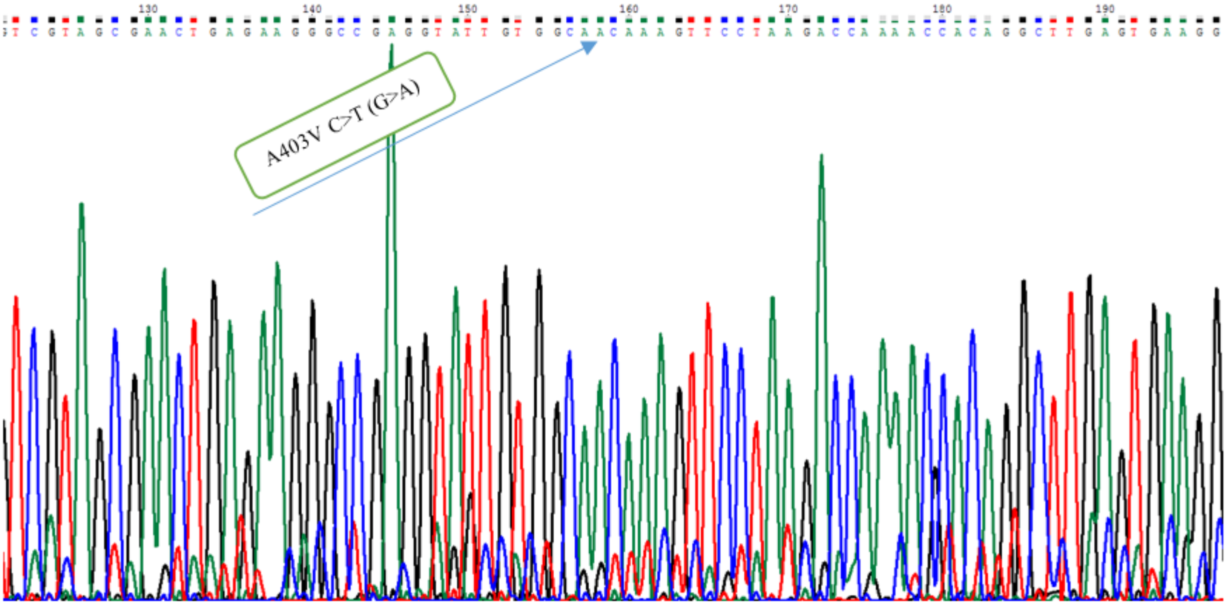

B

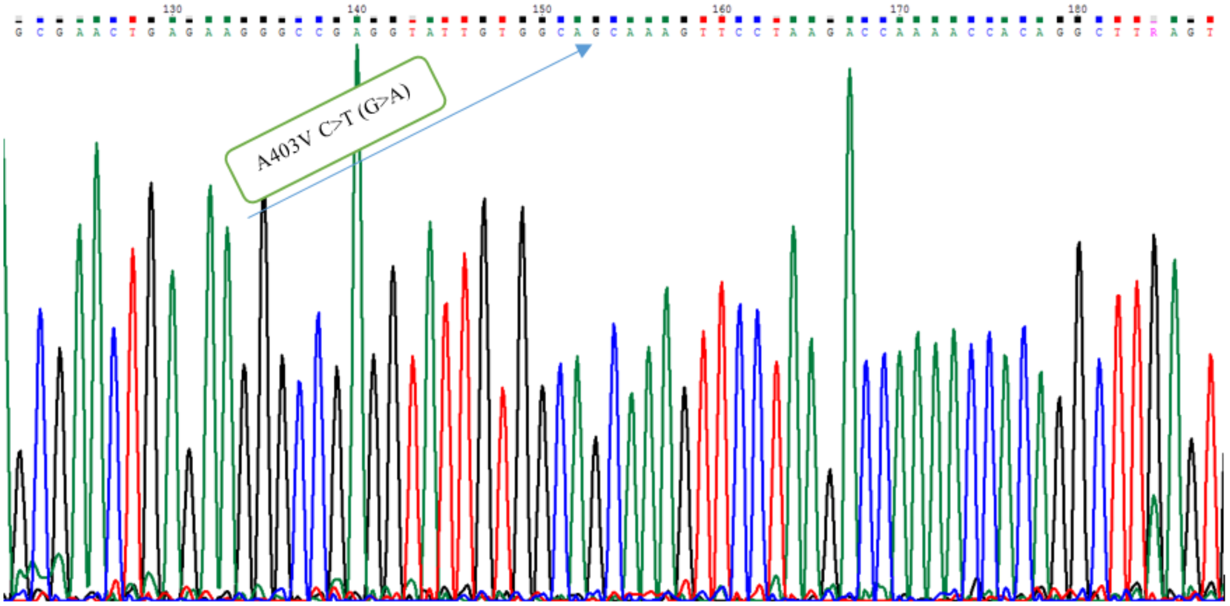

Supplementary Fig. 11. Homozygous (A) mutant and (B) normal for A403V C>T (G>A) (reverse strand).

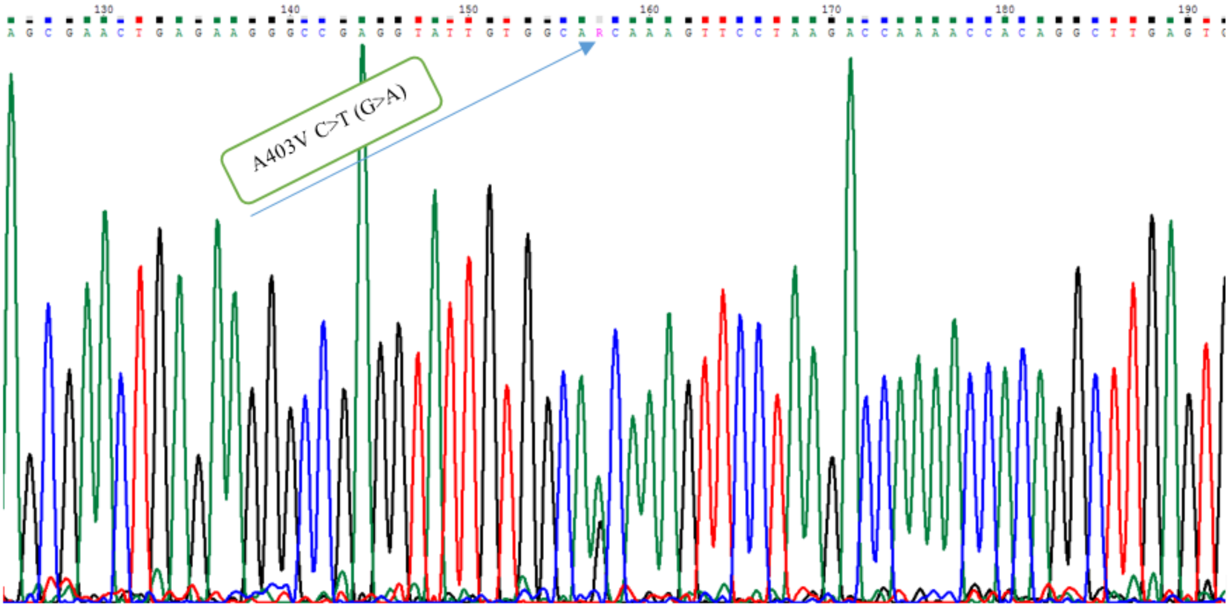

Supplementary Fig. 12. Heterozygous for A403V C>T (G>A) (reverse strand).

A

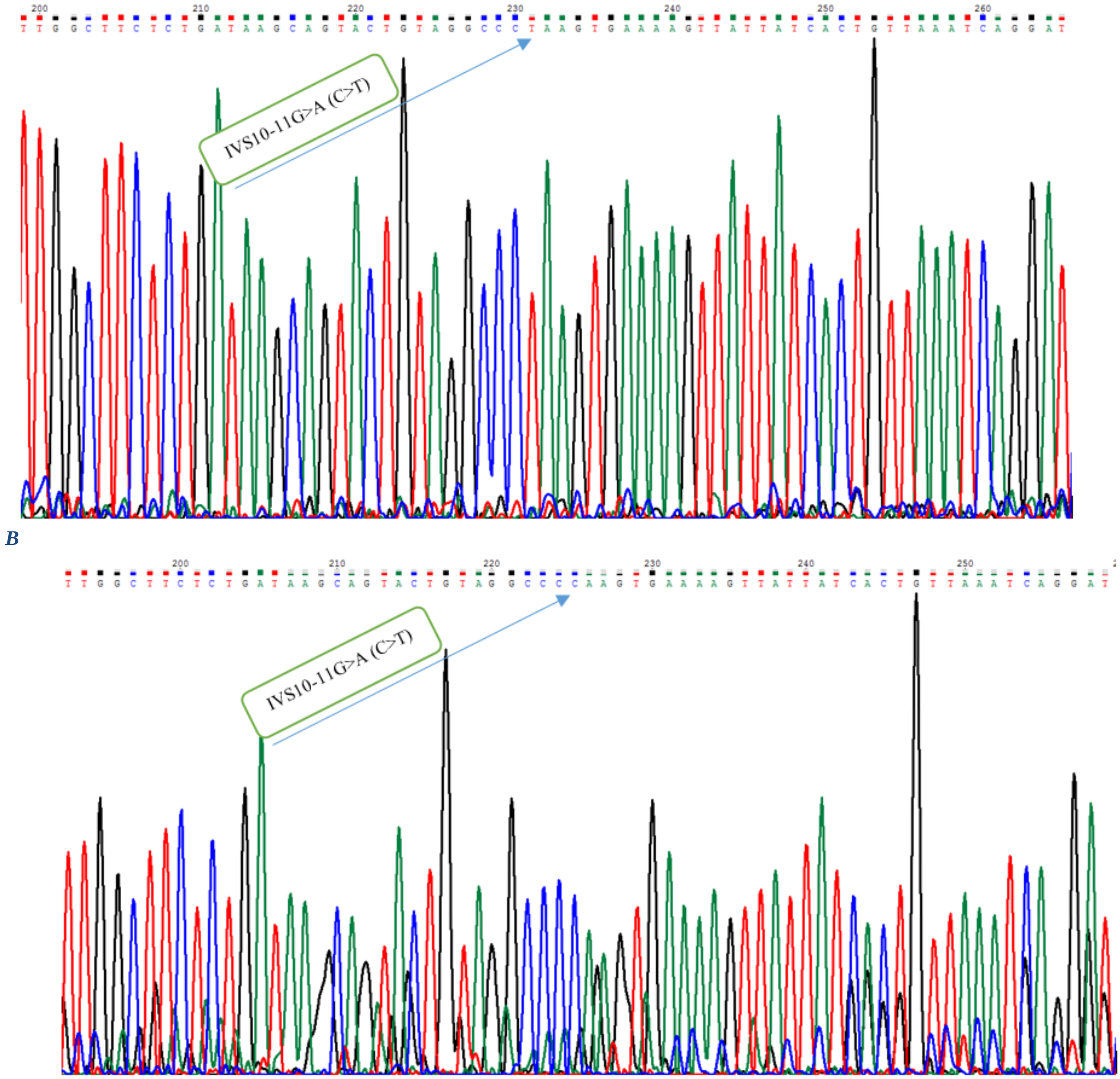

Supplementary Fig. 13. Homozygous (A) mutant and (B) normal for IVS10-11 G>A (C>T) (reverse strand).

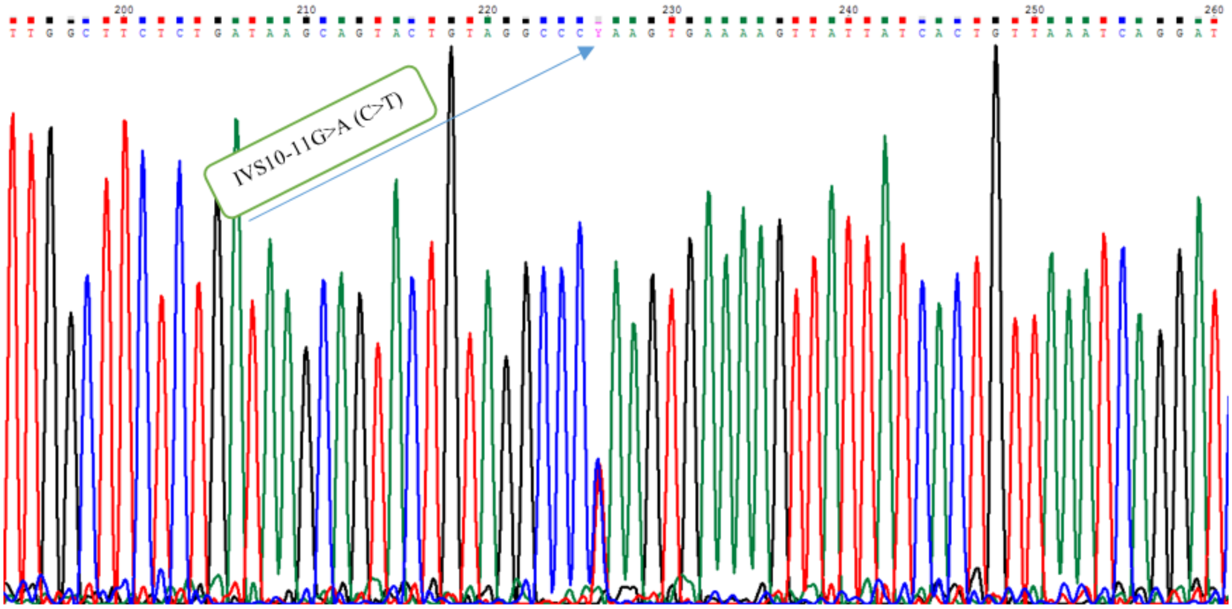

Supplementary Fig. 14. Heterozygous for IVS10-11G>A (C>T) (reverse strand).

A

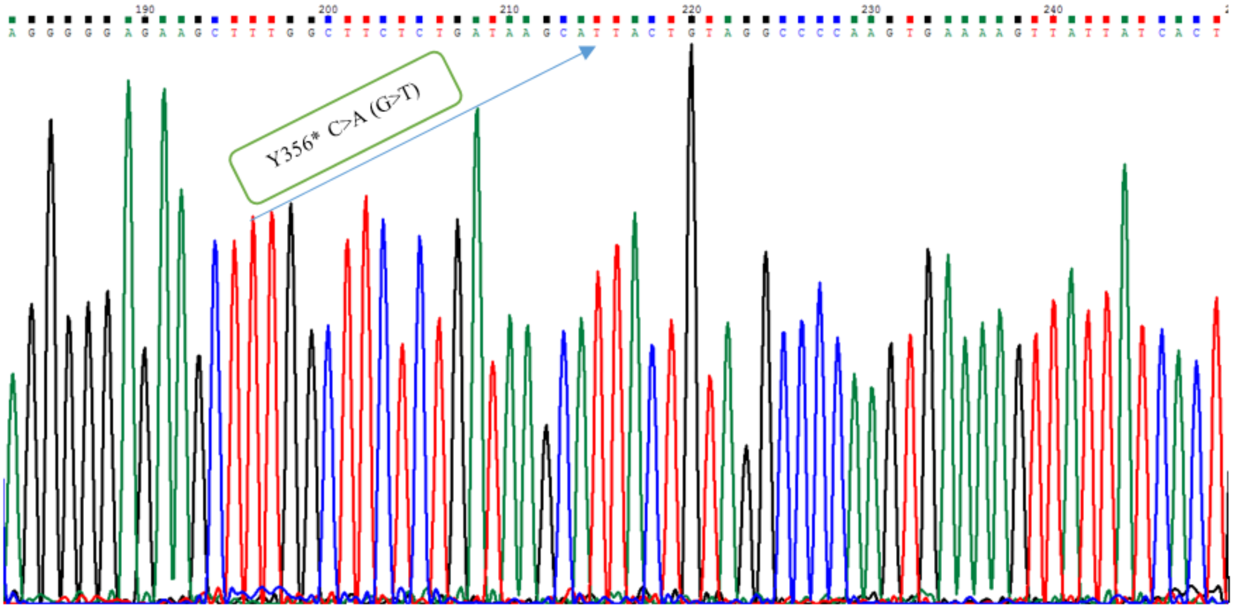

B

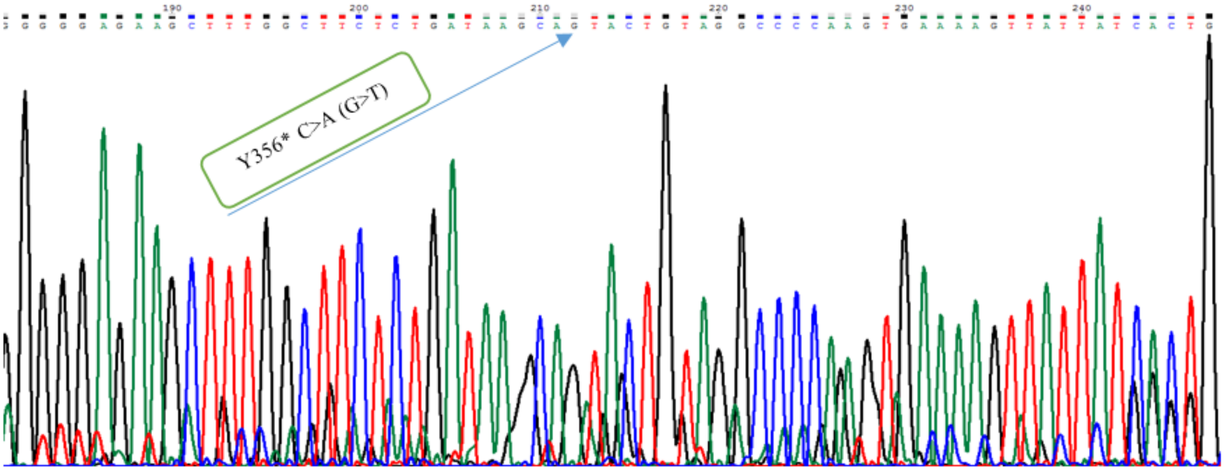

Supplementary Fig. 15. Homozygous (A) mutant and (B) normal for Y356* C>A (G>T) (reverse strand).

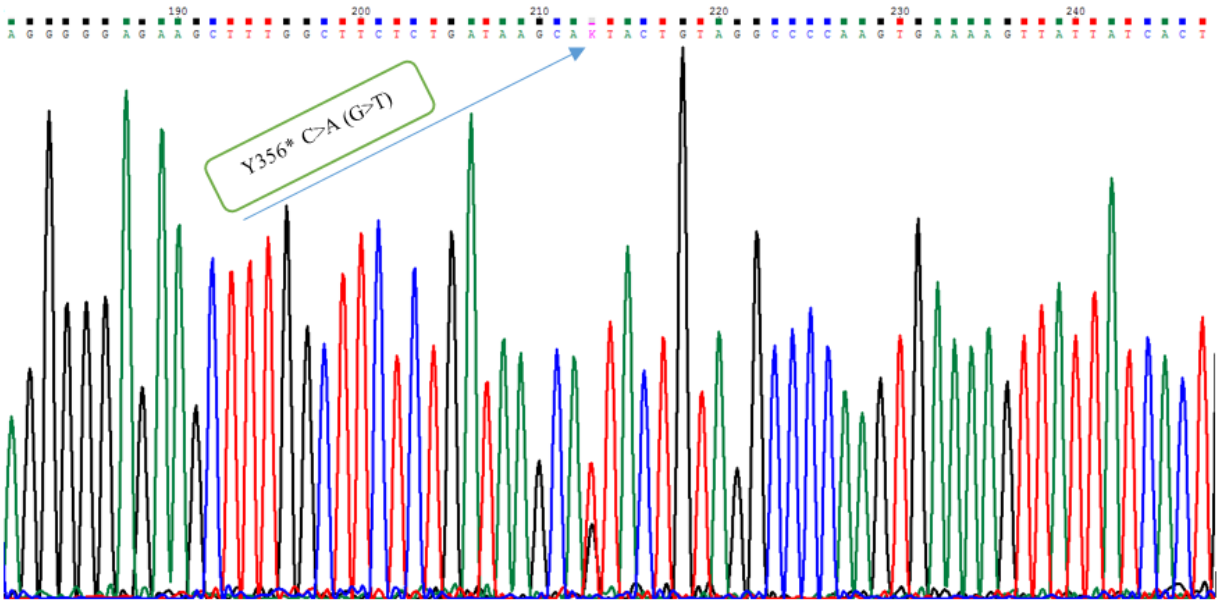

Supplementary Fig. 16. Heterozygous for Y356* C>A (G>T) (reverse strand).

A

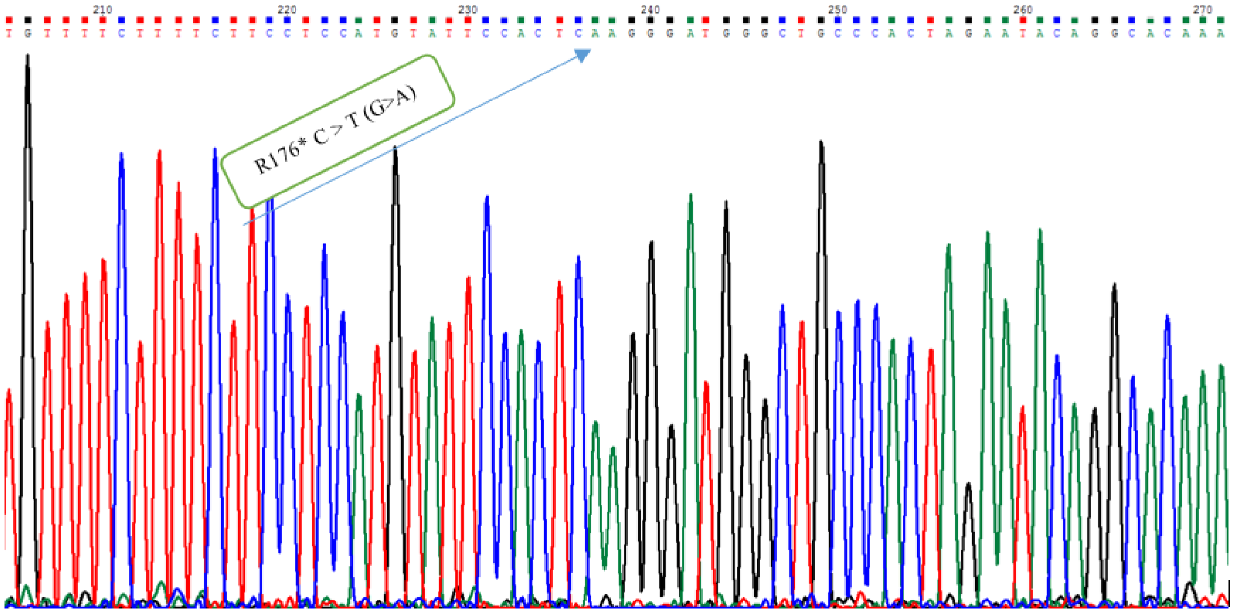

B

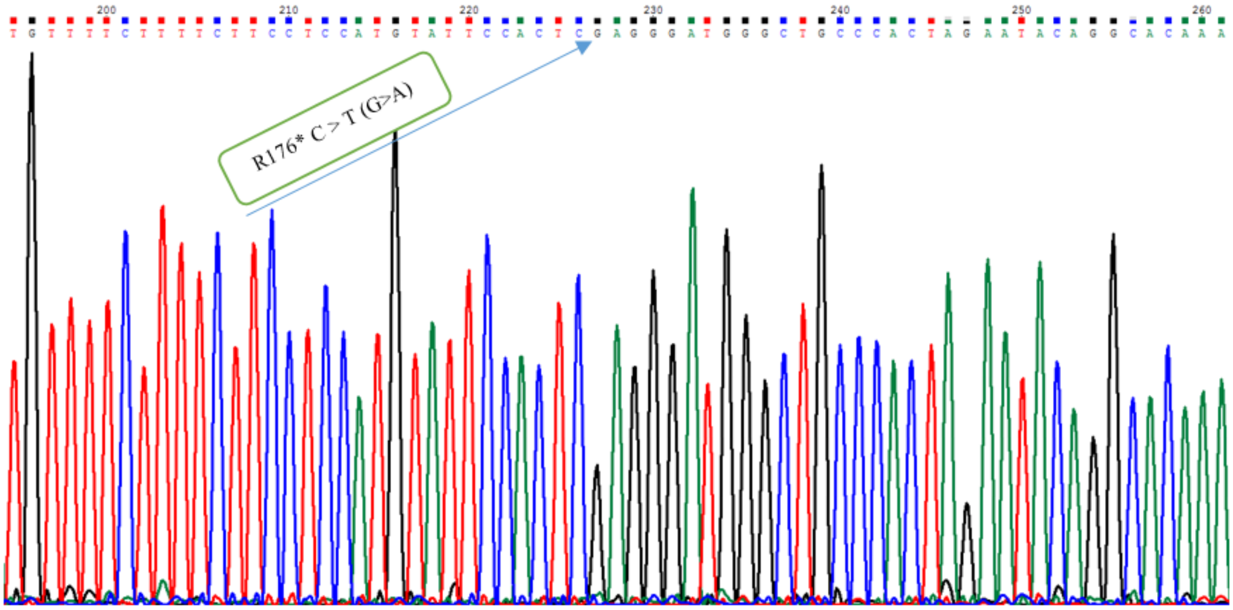

Supplementary Fig. 17. Homozygous (A) mutant and (B) normal for R176* C > T (G>A) (reverse strand).

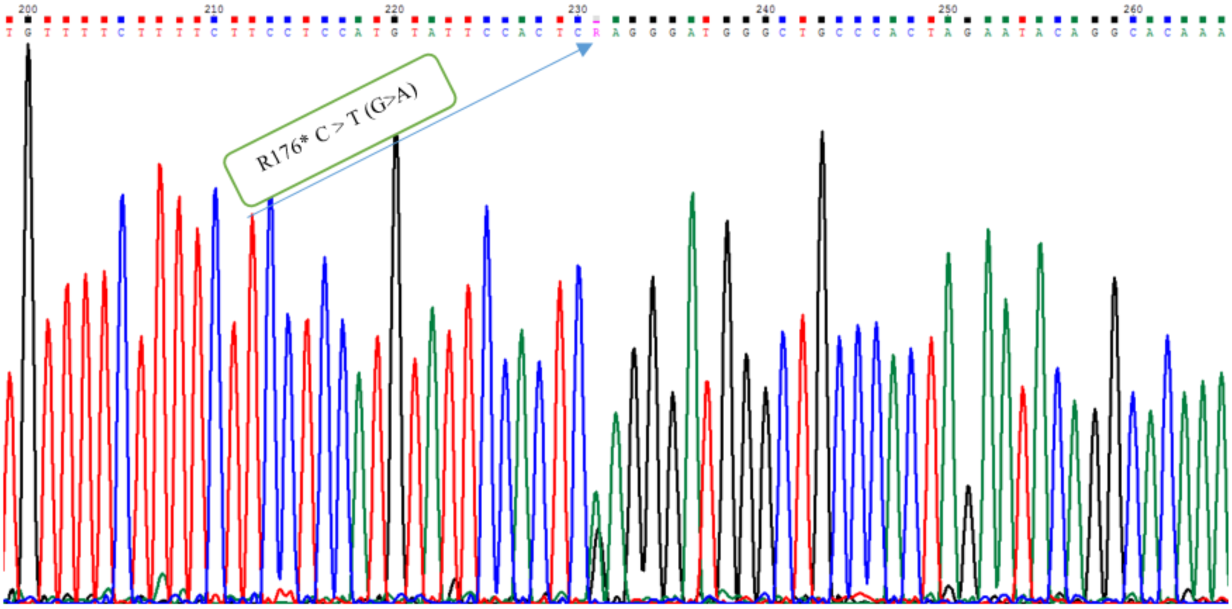

Supplementary Fig. 18. Heterozygous for R176* C > T (G>A) (reverse strand).

A

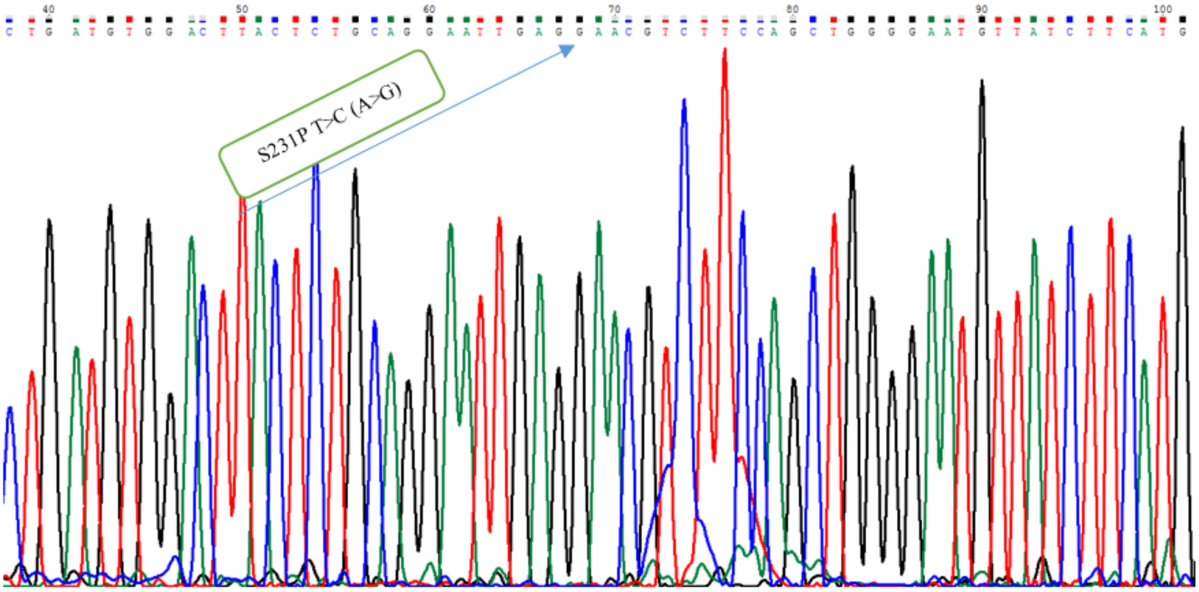

B

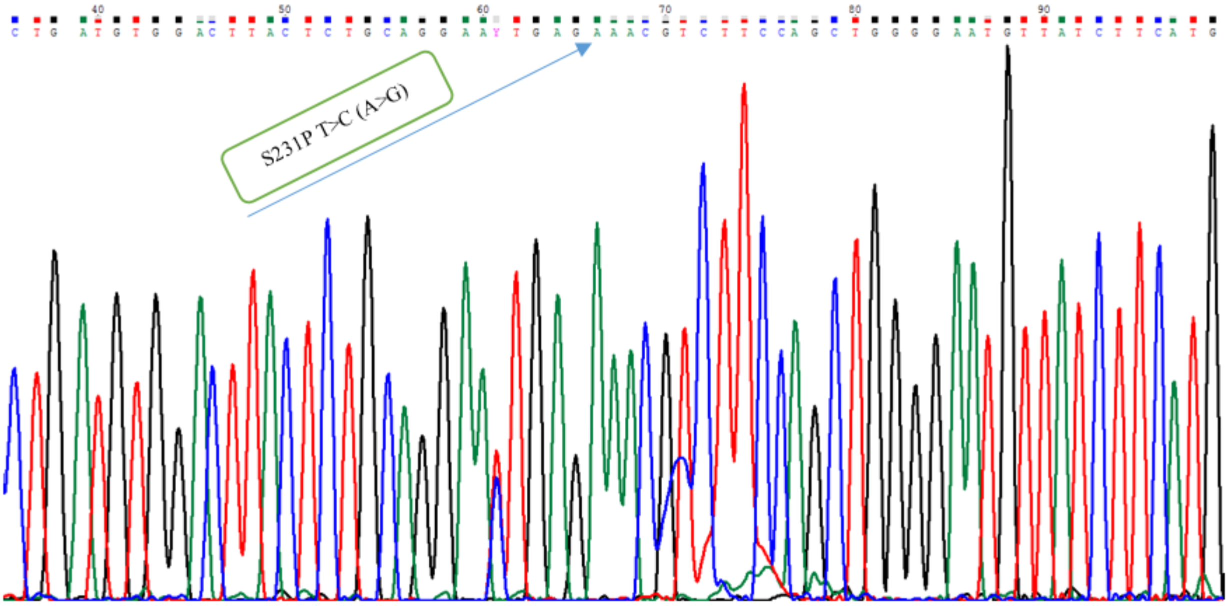

Supplementary Fig. 19. Homozygous (A) mutant and (B) normal for S231P T>C (A>G) (reverse strand).

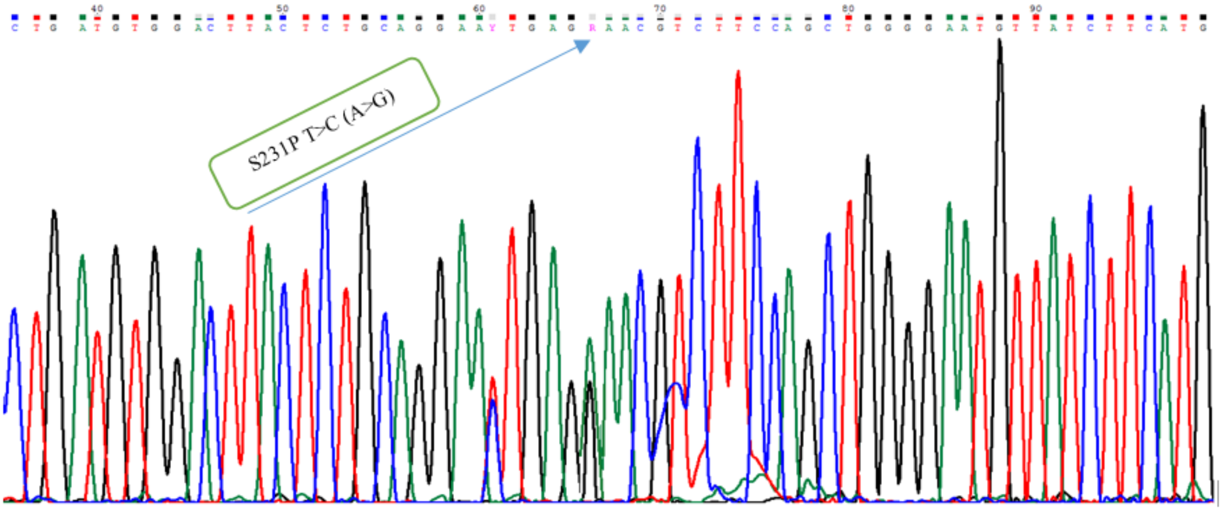

Supplementary Fig. 20. Heterozygous for S231P T>C (A>G) (reverse strand).
